# Conserved meningeal lymphatic drainage circuits in mice and humans

**DOI:** 10.1084/jem.20220035

**Published:** 2022-07-01

**Authors:** Laurent Jacob, Jose de Brito Neto, Stephanie Lenck, Celine Corcy, Farhat Benbelkacem, Luiz Henrique Geraldo, Yunling Xu, Jean-Mickael Thomas, Marie-Renee El Kamouh, Myriam Spajer, Marie-Claude Potier, Stephane Haik, Michel Kalamarides, Bruno Stankoff, Stephane Lehericy, Anne Eichmann, Jean-Leon Thomas

**Affiliations:** 1 Institut du Cerveau, Pitié-Salpêtrière Hospital, Centre National de la Recherche Scientifique, Institut National de la Santé et de la Recherche Médicale, Sorbonne Université, Paris, France; 2 Paris Cardiovascular Research Center, Institut National de la Santé et de la Recherche Médicale, Université de Paris, Paris, France; 3 Biomedical Sciences Institute, Federal University of Rio de Janeiro, Rio de Janeiro, Brazil; 4 Department of Neuroradiology, Pitie-Salpêtrière Hospital, Sorbonne University, Paris, France; 5 Siemens Healthcare SAS, Saint-Denis, France; 6 Department of Internal Medicine, Cardiovascular Research Center, Yale University School of Medicine, New Haven, CT; 7 Department of Cellular and Molecular Physiology, Yale University School of Medicine, New Haven, CT; 8 Department of Neurosurgery, Pitie-Salpêtrière Hospital, Sorbonne University, Paris, France; 9 Department of Neurology, St Antoine Hospital, Assistance Publique Hôpitaux de Paris – Sorbonne, Paris, France; 10 Centre for NeuroImaging Research, Institut du Cerveau et de la Moelle épinière, Paris, France; 11 Department of Neurology, Yale University School of Medicine, New Haven, CT

## Abstract

Meningeal lymphatic vessels (MLVs) were identified in the dorsal and caudobasal regions of the dura mater, where they ensure waste product elimination and immune surveillance of brain tissues. Whether MLVs exist in the anterior part of the murine and human skull and how they connect with the glymphatic system and extracranial lymphatics remained unclear. Here, we used light-sheet fluorescence microscopy (LSFM) imaging of mouse whole-head preparations after OVA-A^555^ tracer injection into the cerebrospinal fluid (CSF) and performed real-time vessel-wall (VW) magnetic resonance imaging (VW-MRI) after systemic injection of gadobutrol in patients with neurological pathologies. We observed a conserved three-dimensional anatomy of MLVs in mice and humans that aligned with dural venous sinuses but not with nasal CSF outflow, and we discovered an extended anterior MLV network around the cavernous sinus, with exit routes through the foramina of emissary veins. VW-MRI may provide a diagnostic tool for patients with CSF drainage defects and neurological diseases.

## Teaser

The present study characterizes the 3D anatomy of meningeal lymphatic vasculature and associated CSF drainage by postmortem light-sheet imaging in mice and by real-time magnetic resonance imaging in humans, demonstrating conserved lymphatic circuitries in contact with dural venous sinuses.

## Graphical Abstract

**Figure:**
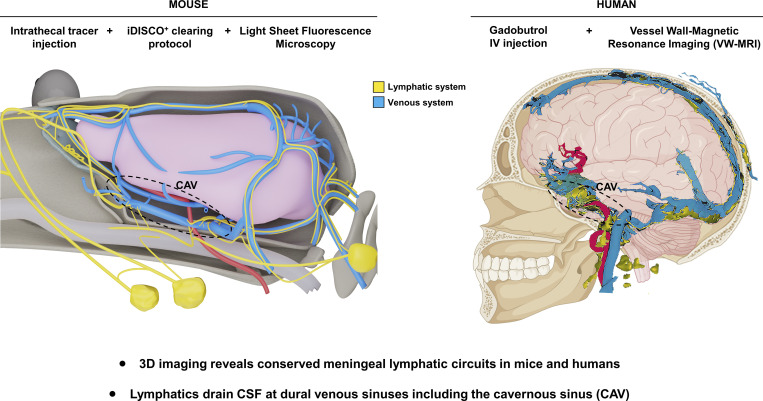

## Introduction

Meningeal lymphatic vessels (MLVs) are located in the dura mater of the brain and spinal cord of various vertebrate species, including humans (Aspelund et al., 2015; Louveau et al., 2015; Absinta et al., 2017; Antila et al., 2017; Jacob et al., 2019). MLVs transport macromolecules and antigens from the cerebrospinal fluid (CSF) and interstitial fluids (ISF) of the central nervous system (CNS) to CNS-draining LNs. By bridging the CNS with the peripheral immune system, MLVs control both brain waste clearance and neuroimmune communication. MLV dysfunction affects various mouse models of neurological disorders, including multiple sclerosis (MS), Alzheimer’s disease, ischemic stroke, intracerebral hemorrhage, traumatic brain injury, and brain tumors (Louveau et al., 2016, 2018; Da Mesquita et al., 2018; Hsu et al., 2019; Esposito et al., 2019; Song et al., 2020; Hu et al., 2020; Bolte et al., 2020; Da Mesquita et al., 2021; Tsai et al., 2022). MLVs have thus become attractive as potential therapeutic targets against CNS pathologies.

The contribution of MLVs to the drainage of CSF outflow from the skull and the clearance of solute waste from CNS tissues has been debated (Louveau et al., 2015; Da Mesquita et al., 2018). Perineural and perivascular spaces were long considered to be the main pathway of CSF outflow into extracranial lymphatics (Tarasoff-Conway et al., 2015; Engelhardt et al., 2016). CSF outflow was shown to drain within cranial nerve sheaths to reach extracranial lymphatics and collecting LNs in the neck (McComb, 1983; Bradbury and Cserr, 1985; Koh et al., 2005). In addition, elegant imaging approaches using near-infrared or dynamic contrast-enhanced magnetic resonance imaging (MRI; Ma et al., 2017; Ma et al., 2019) also showed CSF bulk outflow into extracranial lymphatic pathways that proceeds through the basal cisterns, which are the expansions of the subarachnoid space prolonging around cranial nerves and intracranial vessels (Altafulla et al., 2019). However, an additional contribution of dural lymphatics to CSF drainage was revealed by tracer injections into the CSF, which were taken up by MLVs (Antila et al., 2017; Louveau et al., 2018; Hsu et al., 2019; Ahn et al., 2019). Thus, experimental data support the contribution of MLVs to CSF drainage and warrant a thorough evaluation across the whole dura mater in mice and humans.

The CSF is continuously produced by the choroid plexus and circulates through the CNS internal ventricles, the subarachnoid space, and cisterns, as well as along the perivascular spaces of cerebral arteries and veins (Esposito et al., 2019). CSF exchanges with the ISF of the neuropil through the astroglial glymphatic system, thereby facilitating waste clearance from the CNS (Iliff et al., 2012; Hablitz and Nedergaard, 2021). The glymphatic system generates an outflow of CNS-derived fluids and CSF/ISF waste solutes that subsequently drain out of the skull and the vertebral canal. In the meninges, the glymphatic outflow exits in the perivascular spaces of cerebral veins which converge into the venous sinuses of the dura mater. Dural venous sinuses are key sites of CNS antigen sampling and immune cell egression from the blood into the meninges (Rustenhoven et al., 2021). Dural sinuses also neighbor the MLVs, and the focal ablation of MLVs impaired the glymphatic clearance of toxic protein aggregates in mice (Da Mesquita et al., 2021). MLVs are thus ideally located to collect brain clearance products at exit points of the perivenous spaces, downstream of the glymphatic system. However, lymphatic uptake and CSF/ISF drainage pathways from the dura to the collecting LNs remained to be established.

In fact, MLVs have been less explored for their overall architecture and functional organization than for their physiology and pathophysiology. Murine MLV anatomy has been carefully described in the dura mater of the calvaria (Louveau et al., 2015; Aspelund et al., 2015) and in the posterior fossa of the skull base (Antila et al., 2017; Ahn et al., 2019), identifying lymphatic drainage pathways in the dorsal and caudobasal parts of the skull. Whether additional outflow tracts existed in other parts of the skull remained poorly documented. For example, in the anterior part of the dural sinus system, the cavernous sinus (CAV) has been suggested to participate in CSF absorption (Koh et al., 2005). The CAV collects blood from the superficial and deep middle cerebral veins and the ophthalmic and facial regions (Haines, 2018). CSF drainage from this anterior region has been identified (Antila et al., 2017; Ma et al., 2017; Decker et al., 2021), but the lymphatic circuitry involved remained unknown because the anterior and middle fossae of the skull base are difficult to assess by classic immunohistological techniques. In humans, noninvasive imaging of dural lymphatic is of interest for the diagnosis and prognosis of neurological diseases (Absinta et al., 2017; Ding et al., 2021), and different MRI protocols to detect MLVs have been reported (Absinta et al., 2017; Ringstad and Eide, 2020; Wu et al., 2020; Ding et al., 2021; Albayram et al., 2022).

In this work, we investigated CSF lymphatic drainage with submillimeter resolution by large-field imaging of the whole head using postmortem light sheet fluorescence microscopy (LSFM) imaging in mice (Jacob et al., 2019; Jacob et al., 2020) and improved real-time MRI in humans. Because both techniques preserved the vascular connections between the meninges and the collecting LNs, we were able to establish a three-dimensional (3D) map of the entire lymphatic CSF drainage network in mice and humans. Both approaches demonstrated a similar circa-cerebral MLV architecture and relationship between MLVs and dural venous sinuses, with limited MLV connections with the nasal lymphatic bed, and a conserved pattern of CAV-associated MLVs penetrating the skull through several bilateral foramina of the skull base. Our MRI procedure allowed quantitative mapping of human intra-cranial MLVs and may be relevant for diagnostic imaging of patients with CSF drainage defects and neurological diseases.

## Results

### LSFM 3D imaging of cranial CSF outflow pathways

**Figure 1.**
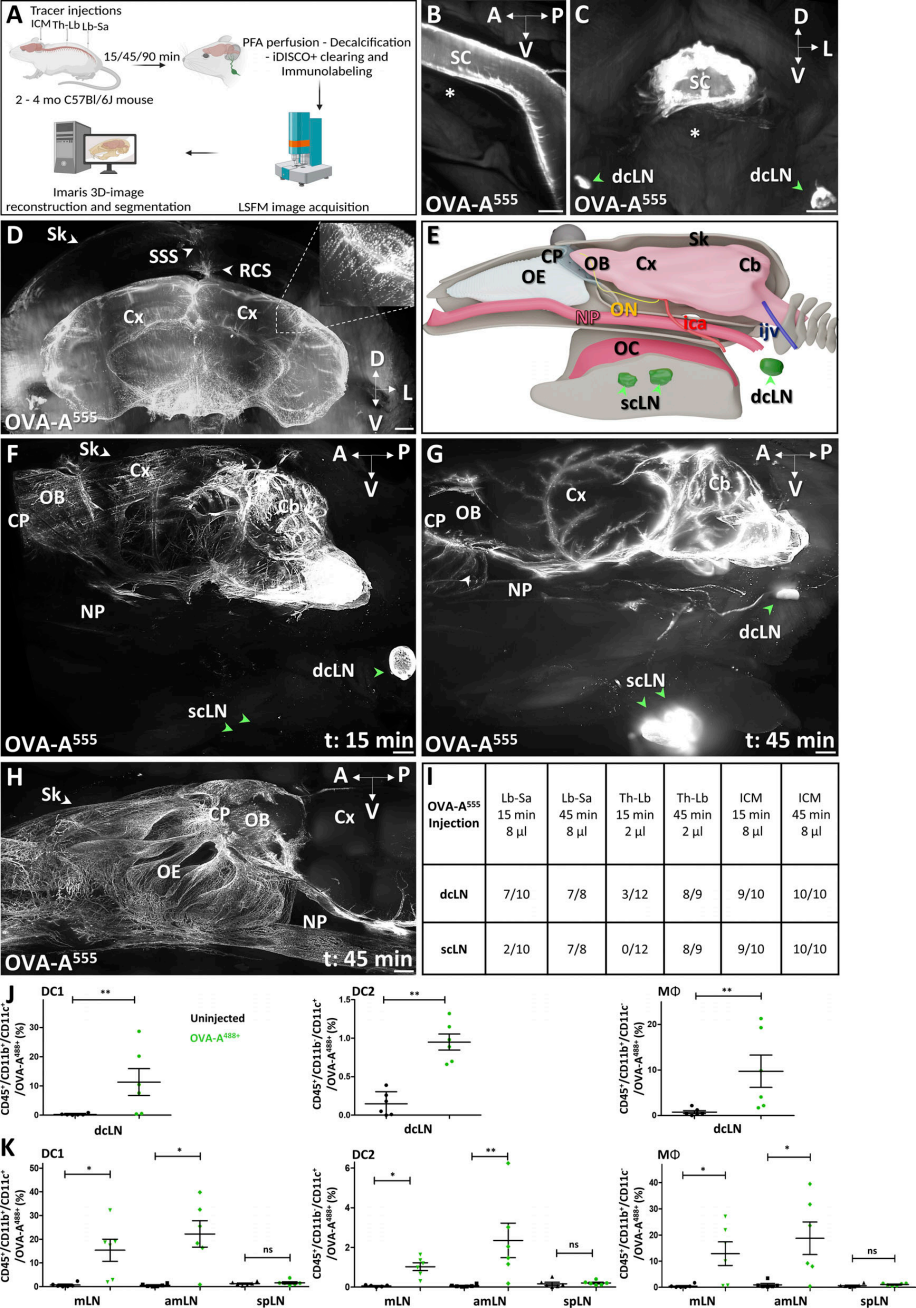
**iDISCO-LSFM imaging of CSF tracer drainage. (A)** Schematic of the experimental workflow. Arrows indicate sites of tracer injection, ICM, Th-Lb, or Lb-Sa. **(B)** LSFM imaging of OVA-A555 (white) in the pia mater of the spinal cord (SC) 90 min after Th-Lb injection. Asterisk, vertebral body. **(C)** Coronal section of cervical SC 45 min after Th-Lb injection shows OVA-A555 in the SC and in the dcLNs (green arrowheads). **(D)** LSFM view of a coronal section of the forebrain shows OVA-A^555^ in phagocytic cells (inset) along the glymphatic perivascular spaces. Note that brain size is reduced by the iDISCO^+^ protocol, but meningeal layers are preserved. The dura and superior sagittal sinus (SSS) are in contact with the skull (Sk), and tracer drainage can be followed through the intact calvaria. OVA-A^555+^ cells are also found along the SSS and the rostral confluence of sinuses (RCS). Cx, cortex. **(E)** 3D schematic of the lateral view of the head. Cb, cerebellum; CP, cribriform plate; ica, internal carotid artery; ijgv, internal jugular vein; NP, nasopharynx; OB, olfactory bulb; OC, oral cavity; OE, olfactory epithelium; ON, optic nerve. Green arrowheads, scLN. **(F–H)** Lateral views of OVA-A^555^ in mice sacrificed 15 min (F) or 45 min (G and H) after Lb-Sa injection. OVA-A^555^–labeled perivascular glymphatic spaces (F and G). OVA-A^555^ labeled the dcLN but not the scLN at 15 min (F, green arrowheads), while both dcLN and scLN are labeled at 45 min (G). Lymphatic afferent vessels extend from the NP toward the LNs (G). **(H)** The same pattern in another mouse with a zoom on the anterior part of the CP and OE. Note OVA-A^555+^ vessels in the OE and around the NP (G and H). **(I)** Summary of tracer injections and number of tracer-positive cervical LNs per mouse. **(J and K)** Quantification of OVA-A^555+^ DC1s (CD45^+^/CD11b^+^/CD11c^+^), DC2s (CD45^+^/CD11b^−^/CD11c^+^), and MΦ (CD45^+^/CD11b^+^/CD11c^−^) among total CD45^+^ cells FACS-sorted from the dcLN, mandibular LN (mLN), accessory mandibular LN (amLN), and superficial parotid LN (spLN) of non-injected mice or mice injected into the Th-Lb spine with OVA-A^488^. *n* = 6 mice/group. Data show mean + SEM; Mann–Whitney *U* test (J) and one-way ANOVA with Dunn’s multiple-comparisons test (K); *, P < 0.05; **, P < 0.01. A, anterior; D, dorsal; L, lateral; P, posterior; V, ventral. Scale bar: 500 μm (B–D and H); 800 μm (F and G).

**Figure S1.**
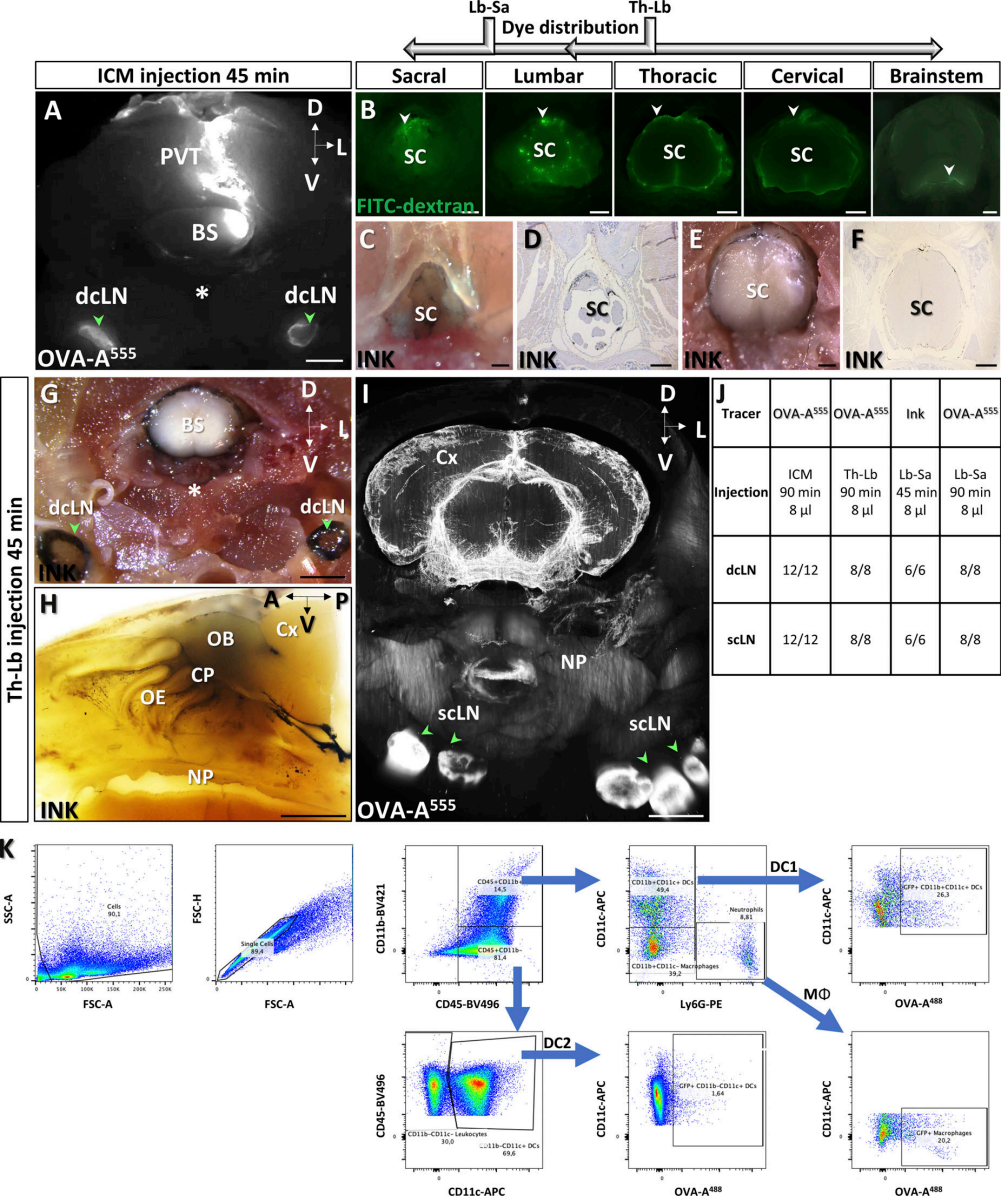
**OVA-A**^**555**^ **tracer distribution after ICM and intraspinal injection. (A)** Coronal section of the cervical spine after ICM injection of OVA-A^555^ (white). Note that OVA-A^555^ spillover labeled paravertebral tissues (PVT, arrowhead) and the brainstem (BS). Asterisk, vertebral body. **(B)** FITC-dextran tracer distribution 45 min after Th-Lb or Lb-Sa injection. Macroscopic imaging of tracer labeling (white arrowheads) along the caudorostral axis of the spine. SC, spinal cord. **(C–F)** Coronal sections of the sacral (C and D) and cervical (E and F) spine showing ink deposits (black) in the meninges along the caudorostral axis. Macroscopic imaging of dissected spine segments (C and E) and bright-field microscopic imaging of paraffin-embedded spine sections (D and F). **(G)** Coronal section of the brainstem (BS) showing ink deposits in the meninges and inside the dcLNs (green arrowheads). Asterisk, vertebral body. **(H)** Sagittal section of the forehead showing ink deposits in the meninges of the olfactory bulb (OB), in the cribriform plate (CP), the olfactory epithelium (OE), and the nasopharynx (NP). Cx, cortex. **(I)** LSFM coronal view of the CSF tracer pattern (OVA-A^555^, white) in the perivascular spaces of the cortex and mesencephalon, and in the scLNs (green arrowheads). **(J)** Table of additional tracer injection experiments showing the number of mice per group with tracer-labeled cervical LNs 45 and 90 min after injection. **(K)** Representative flow dot plots used to separate different populations of OVA^488+^ myeloid cells in cervical LNs in Fig. 1, J and K. A, anterior; D, dorsal; L, lateral; P, posterior; V, ventral. Scale bar: 500 μm (B–F); 2 mm (A and G–I).

We performed LSFM imaging of whole adult mouse head preparations to visualize cranial CSF outflow (Fig. 1 A). Mice were injected with fluorescently tagged Ovalbumin (OVA-A^555^, 2–8 μl per mouse) into the cisterna magna or into the thoracolumbar (Th-Lb) or lumbosacral (Lb-Sa) spinal cord. Whole-head preparations were decalcified and iDISCO^+^-clarified to allow LSFM imaging through the skull and head tissues (Fig. 1 A). As intracranial tracer leakage occasionally occurred after intra–cisterna magna (ICM) injection (Fig. S1 A), we preferentially used intraspinal injections to monitor cranial CSF drainage. OVA-A^555^ deposits were consistently detected along the pia mater and in the perivascular spaces of the spinal cord and brain, as well as within deep cervical LNs (dcLNs; Fig. 1, B and C). A large fraction of the OVA-A^555^ tracer concentrated within phagocytic cells in perivascular spaces of the brain (Fig. 1 D and Video 1). OVA-A^555^ spread cranially and caudally from Th-Lb and Lb-Sa injection sites, and its spatial distribution was similar to that of other tracers such as fluorescent FITC-dextran or India ink (Fig. S1, B–G).

**Video 1.** **OVA-A555 tracer (white) labels phagocytic cells located along perivascular spaces of the brain vasculature as well as along the superior sagittal sinus in the dura mater.** Frame rate, 24 frames/s.

To study the kinetics of CSF drainage from the meninges to collecting LNs, we examined sagittal views of clarified whole head preparations (Fig. 1, E–H). 15 min after intraspinal OVA-A^555^ injection, tracer localized in the perivascular spaces of the cortex and olfactory bulbs as well as in dcLNs (Fig. 1 F). At 45 and 90 min after intraspinal OVA-A^555^ injection, the OVA-A^555^ pattern extended beyond the cribriform plate into the olfactory epithelium, and also labeled the superficial cervical LNs (scLNs; Fig. 1, G and H; Fig. S1, H–J; and Videos 2 and 3). After ICM injection, OVA was already detected in dcLNs and scLNs at 15 min in 90% of mice and at 45 and 90 min in 100% of all cervical LNs (Fig. 1 I), confirming faster drainage from the subarachnoid space in the brain than the caudal spine. However, the distribution of OVA-A^555^ labeling from all three injection sites was similar between mice, and CSF drainage kinetics were similar between injected OVA-A^555^ or ink tracers (Fig. S1 J).

**Video 2.** **OVA-A555 tracer (white) labels brain meninges and, outside of the skull, the nasal cavity, the nasopharynx, and the cervical LNs. **Frame rate, 24 frames/s.

**Video 3.** **OVA-A555 tracer (white) labels the scLNs.** Frame rate, 25 frames/s.

The cellular tracer uptake in the cervical LNs was analyzed by flow cytometry 90 min after Th-Lb injection of OVA-A^488^ (8 μl). LNs were dissected and labeled with a cocktail of antibodies recognizing type 1 dendritic cells (DC1: CD45^+^/CD11b^+^/CD11c^+^), type 2 dendritic cells (DC2: CD45^+^/CD11b^−^/CD11c^+^), and macrophages (MΦ: CD45^+^/CD11b^+^/CD11c^−^) for flow cytometry analysis (Fig. S1 K). Compared with uninjected mice, a significant percentage of OVA-A^488+^ DC1, DC2, and MΦ were detected in dcLNs and in mandibular and accessory mandibular scLNs, but not in parotid scLNs of tracer-injected mice (Fig. 1, J and K), suggesting that this latter group of cervical LNs is not involved in CSF drainage.

### Tracer uptake by MLVs in the calvaria and the posterior fossae of the skull base

**Figure 2.**
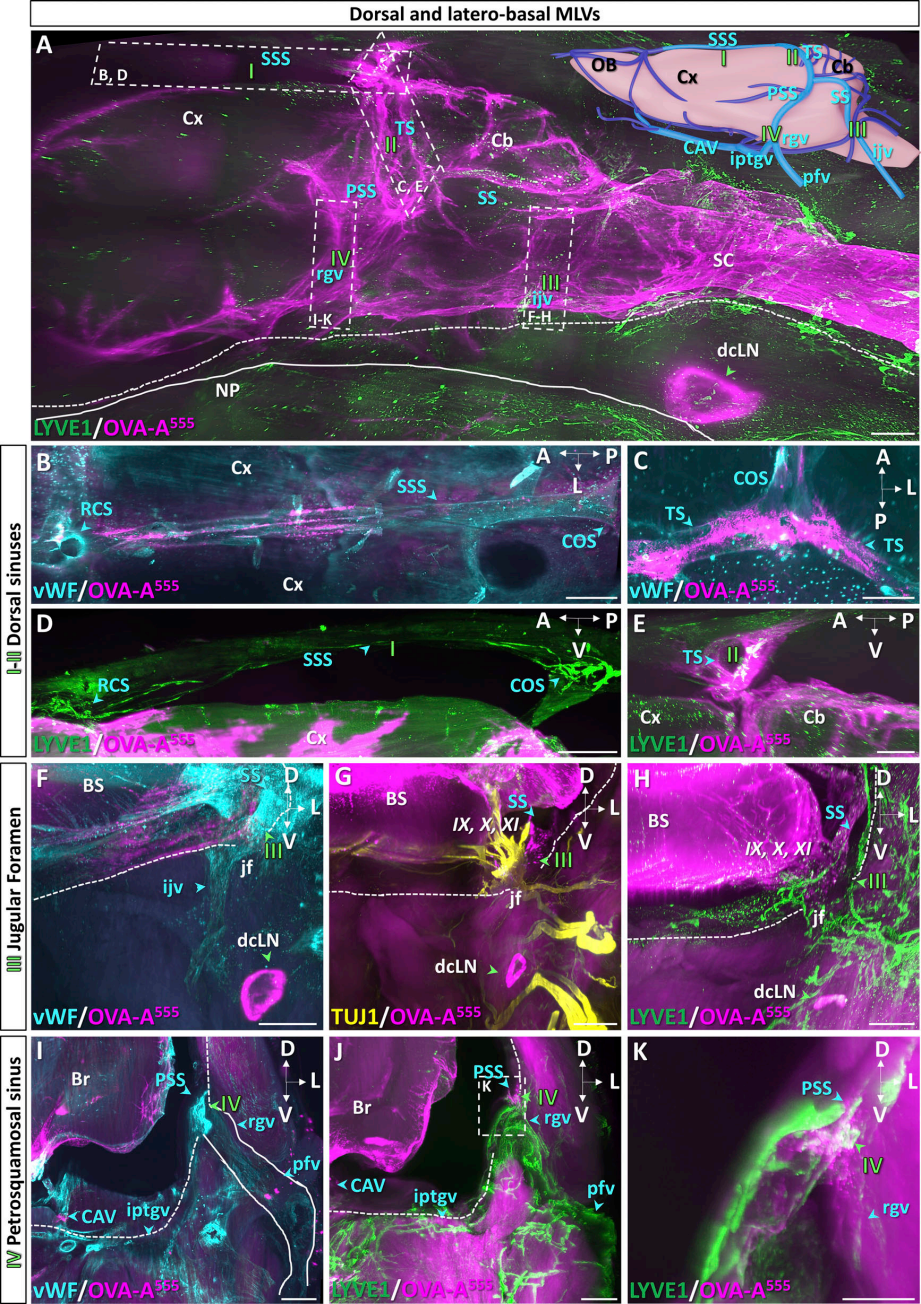
**LSFM imaging of known dorsal and laterobasal MLV drainage pathways. (A)** Lateral view of OVA-A^555^ (magenta) in the posterior head 45 min after Lb-Sa injection labeled with anti-LYVE1 (green). OVA-A^555^ is present in perivascular spaces along the spinal cord (SC), cerebellum (Cb), and cortex (Cx) and in the dcLN (green arrowhead). Roman numbers indicate known MLV circuits: I (SSS), II (transverse sinus TS), III (sigmoid sinus SS and jugular foramen), IV (petrosquamous sinus PSS). NP, nasopharynx (solid line); dashed line, ventral skull border; dashed rectangles, regions magnified in panels I–IV. Inset: Venous sinuses and emissary veins imaged in B–K (light blue) and other venous circuits (dark blue). ijv, internal jugular vein; iptgv, interpterygoid emissary vein; pfv, posterior facial vein; rgv, retroglenoid vein. **(B–E)** Horizontal (B and C) and sagittal (D and E) views labeled with anti-vWF (B and C) and anti-LYVE1 (D and E) antibodies. The SSS (B, blue arrowheads) connects the rostral confluence of sinuses (RCS) with the caudal confluence of sinuses (COS) and the TS (C and E, blue arrowheads). Note discontinuous OVA-A^555^ labeling in the perisinusal spaces (B and C). LYVE1^+^ MLVs along the SSS (D) contain OVA-A^555^ at the TS (white in E). **(F–H)** Coronal views at the level of the jugular foramen (jf) labeled with the indicated antibodies and OVA-A^555^. vWF stained the SS and the jugular vein (ijv, blue arrowhead in F). OVA-A^555^ labels the jf and the dcLN (green arrowheads in F–H). **(G)** TUJ1^+^ cranial nerves exiting the skull through the jf were not colabeled with OVA-A^555^. **(H)** LYVE1^+^ MLVs follow the SS (blue arrowhead) and exit the skull through the jf toward the dcLN. IX, cranial nerve 9 (glossopharyngeal); X, cranial nerve 10 (vagus); X, cranial nerve 11 (spinal accessory); BS, brainstem; dotted line, skull border. **(I–K)** Coronal views at the PSS exit through the skull. **(I)** vWF stains the PSS passing through the petrosquamous fissure (interrupted dashed line) to join the pfv via the rgv. **(J and K)** LYVE1^+^ MLVs follow the PSS, rgv, and pfv. OVA-A^555^ accumulated at the petrosquamous fissure level (J). **(K)** Magnification of the dashed frame in J showing a blind-ended MLV and other OVA-A^555+^/LYVE1^+^ lymphatics (white, green arrowhead). Br, brain. Scale bars: 1,000 μm (A–D); 500 μm (E–J); 250 μm (K).

In all following experiments, OVA-A^555^ (8 μl) was injected into the Lb-Sc spinal cord and mice were sacrificed 45 min later for iDISCO^+^ immunostaining and LSFM imaging of cranial lymphatic drainage. We established a 3D map of dural veins and sinuses (inset in Fig. 2 A and Video 4) using anti–von Willebrand factor (anti-vWF)–immunolabeling overlayed onto the rat dural venous anatomy (Scremin, 2004). MLVs were labeled with anti-LYVE1 antibody. Tracer deposits and MLVs were then localized with respect to the dural veins.

**Video 4.** **3D schematic of adult mouse dural veins and sinuses. **Frame rate, 25 frames/s.

**Figure S2.**
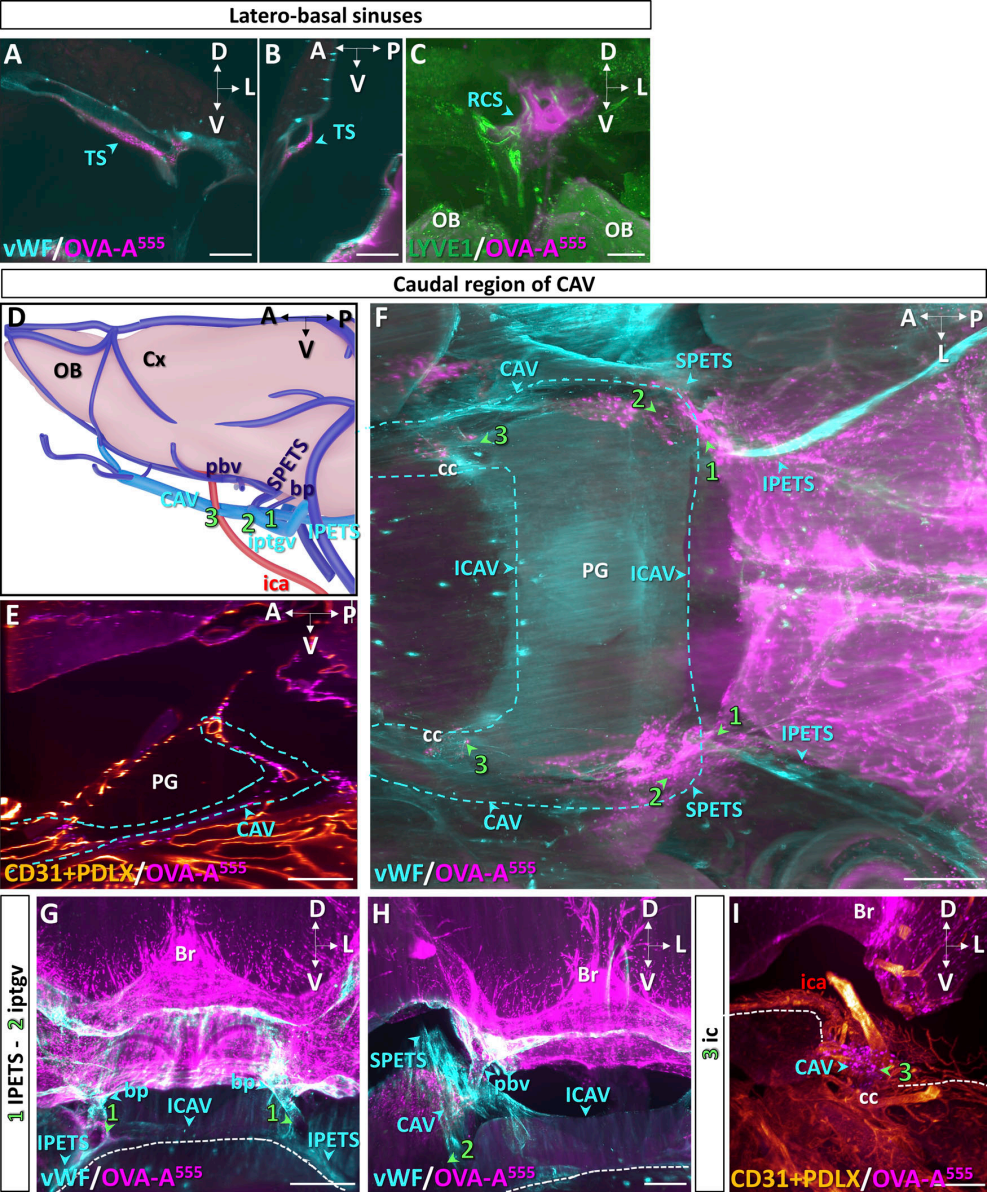
**Perisinusal distribution of CSF tracer and lymphatic uptake hotspots. (A and B)** Coronal (A) and sagittal (B) views of the perisinusal distribution of tracer (OVA-A^555^, magenta, blue arrowheads) along the transversal sinus (TS, vWF^+^, blue). **(C)** LYVE1^+^ MLVs (green) and tracer deposits (magenta) in the rostral convergence of sinuses (RCS). Note tracer accumulation around RCS MLVs (blue arrowhead). OB, olfactory bulb. **(D)** Schematic lateral view of dural venous sinuses and localization of lymphatic uptake hotspots (1–3) in the caudal portion of CAV. bp, basilar plexus; Cx, cortex; ica, internal carotid artery; IPETS, inferior petrosal sinus; iptgv, interpterygoid emissary vein; pbv, posterior basal vein; SPETS, superior petrosal sinus. **(E)** Sagittal view of CD31^+^/PDLX^+^ blood vessels (red) and OVA-A^555^ deposits (magenta) around the pituitary gland (PG) and the CAV (blue dashed line). **(F–I)** Localization of lymphatic uptake hotspots (1–3) in the caudal portion of CAV: horizontal (F) or coronal (G–I) views of tracer deposits (magenta) and vWF^+^ dural sinuses and veins (blue). (I) Blood vessels (CD31^+^/PDLX^+^, orange) at the carotid canal (cc). Dotted line, skull border; ICAV, inter-CAV sinus. Scale bar: 200 μm (A–C); 500 μm (E–I).

In Fig. 2 A, a sagittal view of the caudal part of the head shows the patterns of both OVA-A^555^ deposits and MLVs. In the dorsal region, the tracer was discontinuously distributed along the vWF^+^ sagittal (Fig. 2 B) and transverse sinuses (Fig. 2 C) and accumulated in the perisinusal spaces around the transverse sinus (Fig. S2, A and B). MLVs followed the sagittal and transverse sinuses (Fig. 2, D and E). We detected OVA-A^555^ within LYVE1^+^ vessels at the transverse sinuses and close to LYVE1^+^ MLVs at the rostral confluence of sinuses (Figs. 2 E and S2 C).

The foramen of the jugular vein, which exits the basal skull along with cranial nerves IX, X, and XI (Fig. 2, F and G), displayed a dense network of dural MLVs in contact with the sigmoid sinus, including LYVE1^+^/OVA-A^555+^ capillaries (Fig. 2 H). Sigmoid MLVs exited the skull through the jugular vein foramen and connected to the peripheral lymphatic network that drains into the dcLN (Fig. 2, F–H). A similar pattern of MLVs was observed on the inner side of the petrosquamous fissure, in contact with the petrosquamous sinus (Fig. 2, I–K). Petrosquamous MLVs exited the skull along the retroglenoid vein, then the posterior facial vein (Fig. 2, I and J). In summary, 3D LSFM imaging confirmed the MLV pattern and the localization of tracer uptake hotspots previously characterized on skull cap preparations and sections (Aspelund et al., 2015; Louveau et al., 2015; Antila et al., 2017; Ahn et al., 2019).

### Lymphatic circuits of CAV in the middle fossae of the skull base

**Figure 3.**
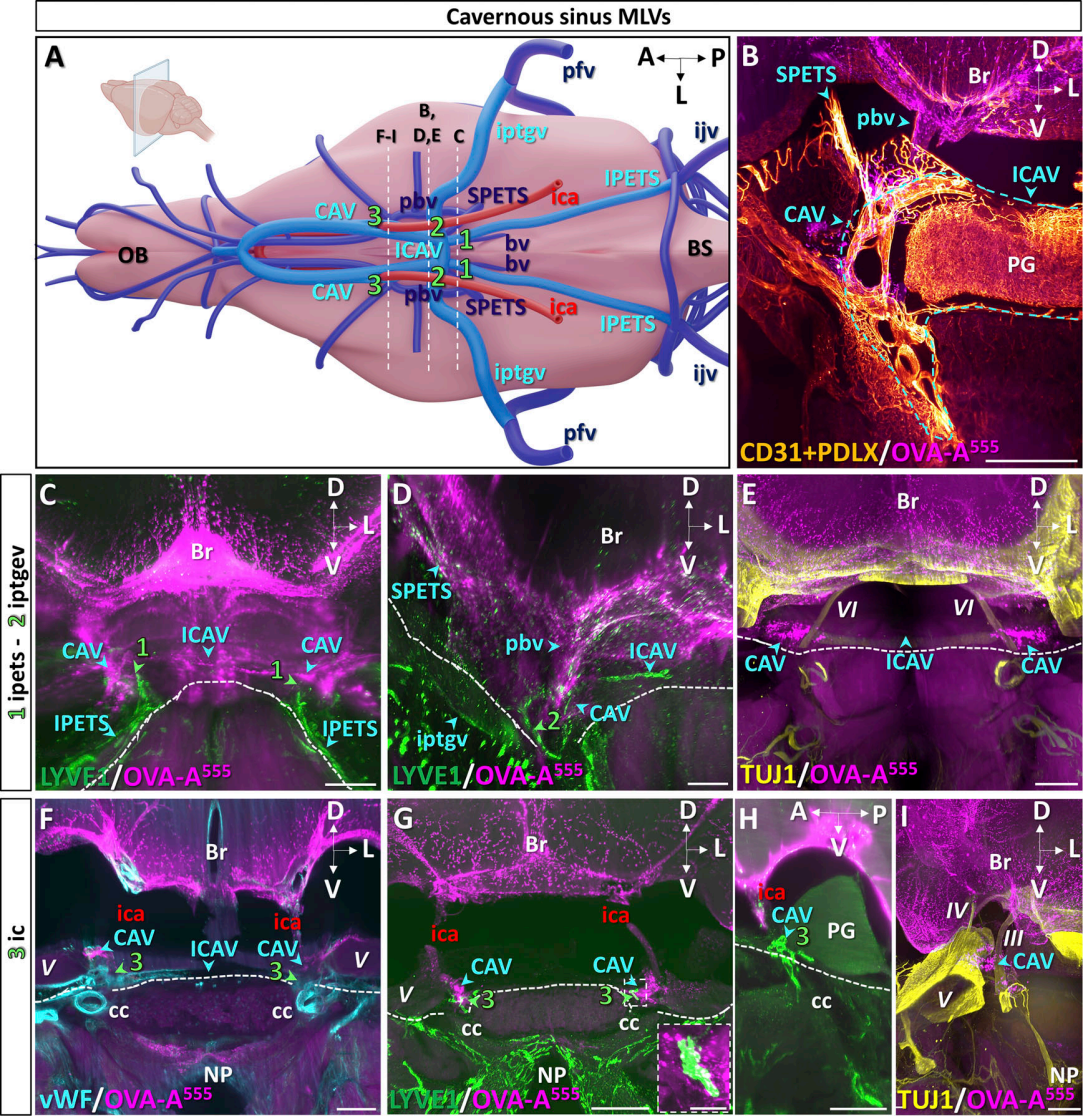
**MLV drainage from the caudal CAV. (A)** Schematic of venous sinuses, veins, and internal carotid artery at the base of the brain. **(B–I)** Dashed lines indicate the level of coronal sections in B–I (inset in A), and lymphatics are numbered 1–3. BS, brainstem; bv, basilar vein; ica, internal carotid artery; ICAV, inter-CAV; ijv, internal jugular vein; IPETS, inferior petrosal sinus; iptgv, interpterygoid emissary vein; OB, olfactory bulb; pbv, posterior basal vein; pfv, posterior facial vein; SPETS, superior petrosal sinus. **(B)** Coronal view of the right CAV. OVA-A^555^ was injected into the Th-Lb spine 45 min before sacrifice, and the sample was labeled with antibodies recognizing blood endothelium markers CD31 and PDLX that label pituitary gland (PG) vessels as well as dura mater veins and sinuses (blue arrowheads). Blue dashed line, limits of CAV and iCAV. Note tracer deposits (magenta) around the CAV and pbv as well as in the leptomeninges. Br, brain. **(C–E)** Cavernous MLVs 1 and 2. Lymphatic vasculature (LYVE1^+^, green in C and D) and TUJ1^+^ cranial nerves (yellow in E) at coronal levels 1 and 2 (green arrowheads in C and D). Note MLVs close to the OVA-A^555+^ cavernous perisinusal space (C) and surrounding the foramen of iptgv (D). Cranial nerves were devoid of tracer deposits (E). VI, cranial nerve 6 (abducens); dotted line, skull border. **(F–I)** Cavernous MLVs 3. Dural veins (vWF, blue in F), lymphatic vasculature (LYVE1^+^, green in G and H), and cranial nerves (TUJ1^+^, yellow in I) at coronal (F, G, and I) and sagittal (H) level 3 (green arrowheads in F–H). MLVs contact the cavernous perisinusal space (G and H) and uptake tracer at the intersection of CAV with internal carotid arteries (white in G, magnified in inset). Cranial nerves were devoid of tracer deposits and MLVs (I). III, cranial nerve 3 (oculomotor); IV, cranial nerve 4 (trochlear); V, cranial nerve 5 (trigeminal); A, anterior; cc, carotid canal; D, dorsal; L, lateral; NP, nasopharyngeal cavity; P, posterior; V, ventral. Scale bar: 500 μm (B–I), 100 μm (inset in G).

We then imaged lymphatic drainage of the skull base in the region of the CAV (Fig. 3 A). Whole-head preparations were labeled with antibodies recognizing vWF to identify veins and with pan-endothelial markers CD31 and podocalyxin (PDLX). We located the CAV caudally on both sides of the pituitary gland and its connection by the inter-CAV (Fig. 3 B and Fig. S2, D–F). The CAV communicates with the jugular veins via the inferior petrosal sinuses (Fig. 3, A and C; and Fig. S2 G) and with the transverse sinuses via the superior petrosal sinuses (Fig. 3, A and D; and Fig. S2 H). They collect blood of the basilar venous plexus and the posterior basal veins and are crossed by the internal carotids (Fig. 3, A, B, and D; and Fig. S2, D and G–I).

Three lymphatic foci were identified in the caudal portion of the CAV (numbered 1–3 in Fig. 3 A). MLVs were detected along the inferior petrosal sinus (1 in Fig. 3, A and C) as well as along the inter-CAV and at the foramen of the inter-pterygoid emissary veins (2 in Fig. 3, A and D). The third group of MLVs (3) concentrated at the intersection of CAV with internal carotid arteries and showed LYVE1^+^ MLVs containing OVA-A^555^ deposits (Fig. 3, F–H; Fig S2 I; and Video 5). In contrast, OVA-A^555^ deposits were not detected along neighboring cranial nerves (VI in Fig. 3 E and III, IV, and V in Fig. 3 I).

**Video 5.** **LYVE1**^**+**^ **MLVs (green) localized at the carotid foramen and associated with perisinusal deposits of OVA-A555 around the CAV. **Frame rate, 24 frames/s.

**Figure 4.**
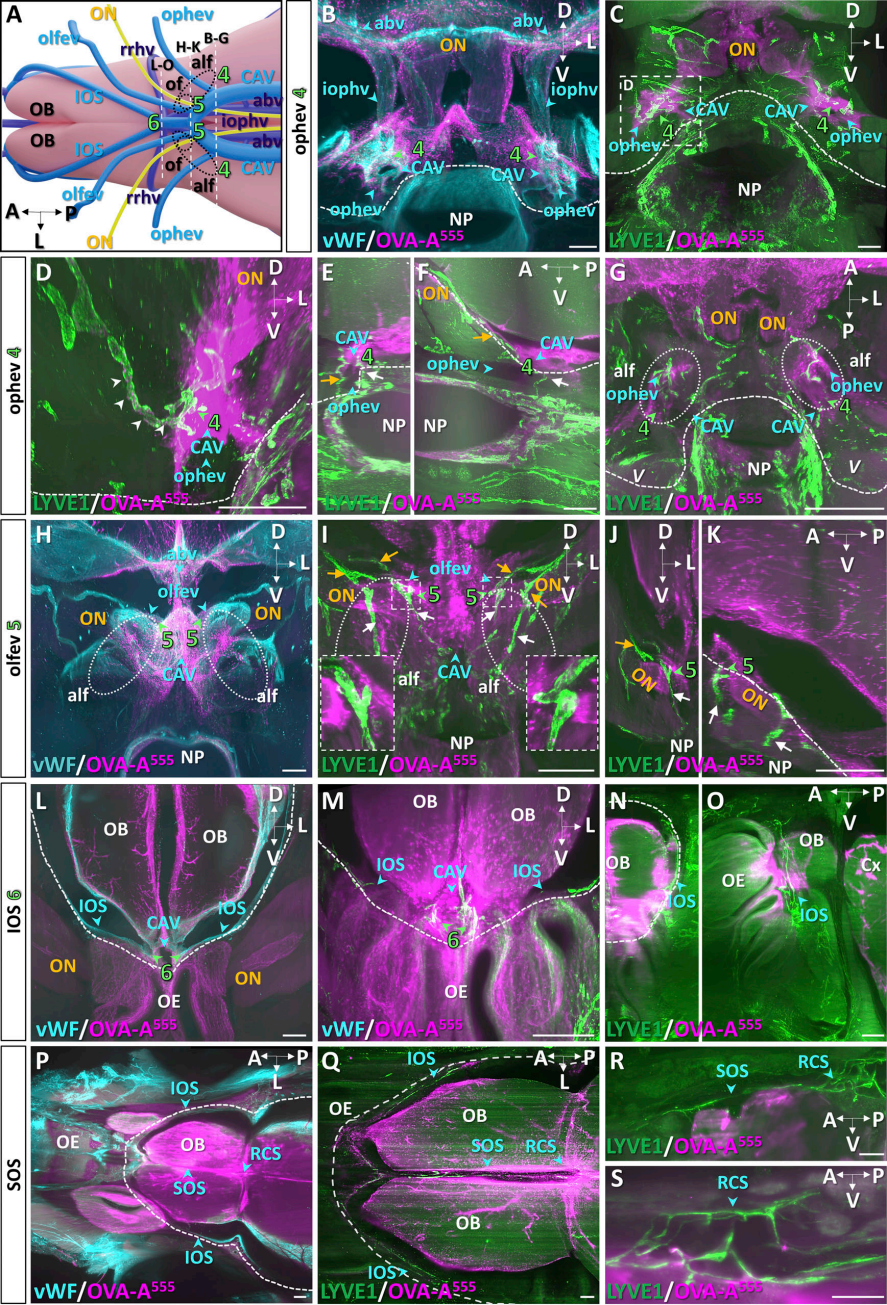
**MLV drainage from the rostral CAV. (A)** Schematic of veins of the ventral forebrain and olfactory bulbs (OB). **(B–O)** Dashed lines indicate the level of coronal sections shown in B–O, and lymphatics are numbered 4–6. Black circles, optic nerve foramen (of); anterior lacerated foramen (alf). abv, anterior basal vein; iophv, internal ophthalmic vein; IOS, inferior olfactory sinus; olfev, olfactory emissary vein; ON, optic nerve (yellow); ophev, ophthalmic emissary vein; RCS, rostral confluence of sinuses; rrhv, rostro-rhinal vein; SOS, superior olfactory sinus. **(B)** vWF^+^ veins (blue) and tracer deposits (OVA-A^555^, magenta) in the iophv perivenous area and around the ophev at lymphatic uptake site 4. Dotted line, skull border. **(C–G)** LYVE1^+^ MLVs (green) and OVA-A^555^ (magenta) around the CAV at level 4 (green arrowheads). Th-Lb (B–F) or ICM (G) injections of OVA-A^555^ were performed 45 min before sacrifice. MLVs contact perisinusal OVA-A^555^ deposits (C). **(D)** Magnification of dotted frame in C. OVA-A^555^ is present within MLVs (white arrowheads). **(E and F)** Coronal (E) and sagittal (F) views of MLVs following the ophev toward the orbital cavity (orange arrows) and connecting ventrally to lymphatics of the nasopharynx (NP, white arrows). **(G)** Note similar labeling pattern to C; MLVs exit the skull through the alf and prolong along the ophev toward the orbital cavity. **(H–K)** Meningeal veins (vWF^+^, blue in H), lymphatic vasculature (LYVE1^+^, green in I–K), and tracer deposits (OVA-A^555^, magenta) on coronal (H–J) and sagittal (K) views at section level 5. **(H)** Tracer accumulated around the confluence of the olfev with the CAV (blue arrowheads). Stippled area, alf. **(I–K)** LYVE1^+^ MLVs follow the olfev to exit the skull toward the orbital cavity (orange arrows) and extend ventrally toward the NP (white arrows). OVA-A^555^ deposits were found in LYVE1^+^ MLVs (insets in I). Coronal (J) and sagittal (K) view of the orbital cavity. MLVs exit the skull toward the orbital cavity (orange arrows) and the NP (white arrows). **(L–O)** Rostral end of CAV at section level 6. **(L)** Tracer accumulated around the confluence of the IOS with the CAV (blue arrowheads). Green arrowheads, MLVs at the rostral end of CAV take up tracer (white in M). MLVs extend rostrally from the CAV along the IOS on the lateral side of each olfactory bulb (N and O, blue arrowhead). OE, olfactory epithelium. **(P–S)** Horizontal (P and Q) and sagittal (R and S) views of the anterior part of the head showing the IOS, SOS, and RCS. Dural veins (vWF^+^, blue in P), lymphatic vasculature (LYVE1^+^, green in Q–S), and tracer deposits (OVA-A^555^, magenta). **(Q)** MLVs connect the CAV with the RCS via the IOS and SOS. **(R and S)** OVA-A^555^ is accumulated around MLVs at the SOS (Q and R) and RCS (R and S) Scale bar: 300 μm (C–S).

Three additional MLV foci were observed in the rostral part of the CAV (numbered 4–6 in Fig. 4 A). MLV foci number 4 and 5 located at the confluence with the ophthalmic and olfactory emissary veins, respectively (Fig. 4, B and H). The OVA-A^555+^ perisinusal area harbored bilateral LYVE1^+^/OVA-A^555+^ foci (Fig. 4, C–G and I–K; and Video 6), including lymphatic capillaries that contained phagocytic cells engaged in uptake of OVA-A^555^ (Fig. 4, D and I), although no free OVA-A^555^ was detected inside lymphatic vessels. LYVE1^+^/OVA-A^555+^ vessels of the CAV exited the skull by the anterior lacerated fissure toward the lymphatic beds of the orbital cavity and nasopharynx (Fig. 4, A, E–G, and I–K). The nasopharyngeal lymphatics were recently shown to mediate extracranial drainage of CSF outflow (Decker et al., 2021).

**Video 6.** **Entry point of the ophthalmic emissary vein into the anterior CAV.** LYVE1^+^ capillaries (green) contact the OVA-A555^+^ perisinusal area of the CAV (magenta) and transport OVA-A555^+^ phagocytic cells. Frame rate, 25 frames/s.

In the most rostral part of the CAV, the sixth site of LYVE1^+^/OVA-A^555+^ capillaries was found near the inferior olfactory sinuses (Fig. 4, A and L–O). These cavernous lymphatic capillaries were prolonged by dural lymphatics running along the superior olfactory sinus toward the dorsal surface of olfactory bulbs and the rostral confluence of sinuses (Fig. 4, P–S).

### Facial lymphatic drainage from ethmoidal and orbito-nasal regions

**Figure 5.**
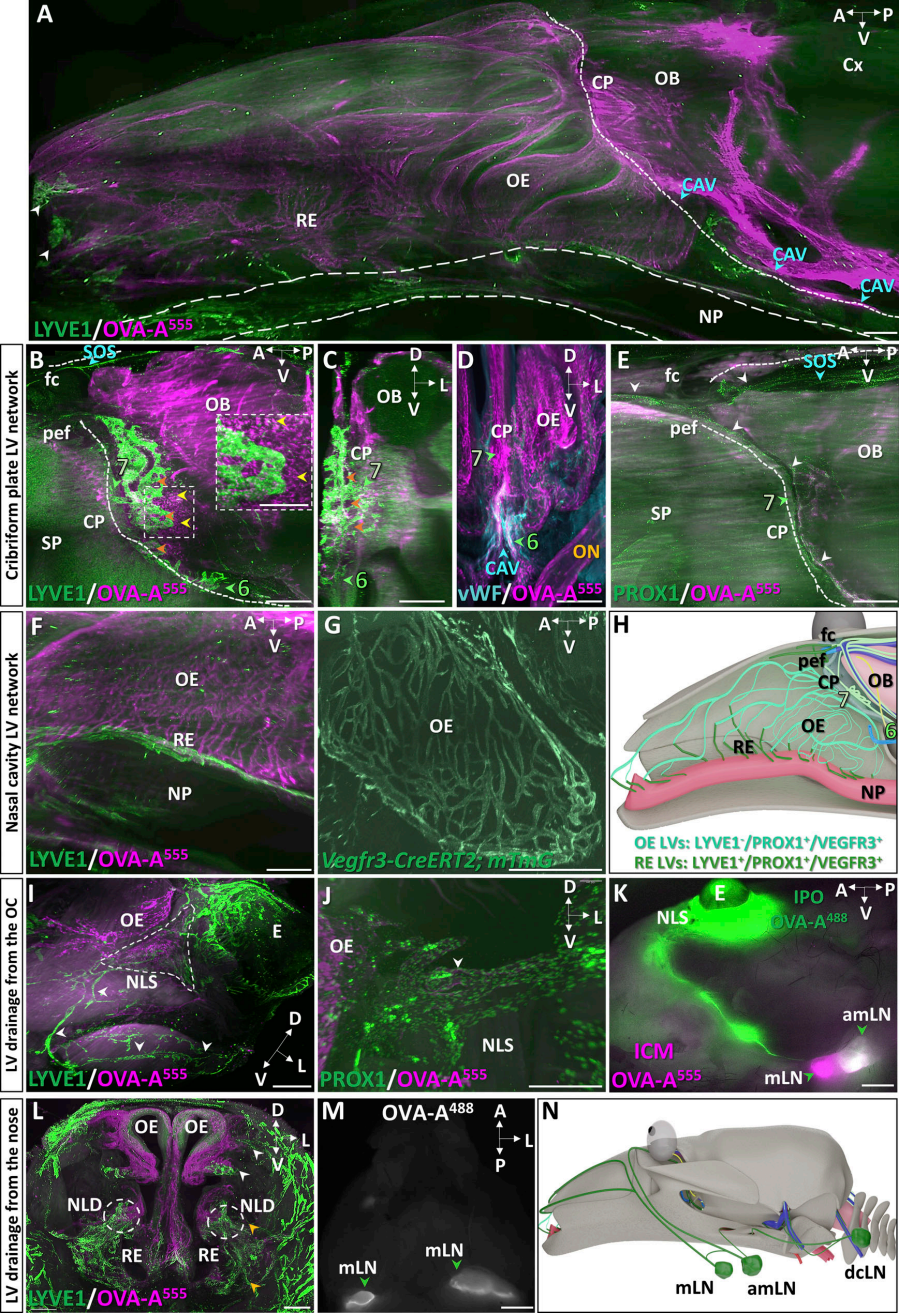
**CSF drainage from the cribriform plate into the nasal cavity. (A)** Sagittal view of the nasal cavity and forebrain 45 min after Th-Lb injection of OVA-A555 (magenta) and labeled with LYVE1 (green). Tracer deposits were detected in meningeal perivascular spaces, the cribriform plate (CP), and throughout the olfactory epithelium (OE) and the respiratory epithelium (RE) of the nasal cavity. Dotted line, limit between intra and extracranial regions; dashed lines, nasopharynx (NP); blue arrowheads, CAV; OB, olfactory bulb. **(B–E)** Lymphatics of the cribriform plate. Lymphatic vasculature (LYVE1^+^, green in B and C; PROX1^+^, green in E), meningeal veins (vWF, blue in D), and tracer deposits (magenta) on sagittal (B and E) and coronal (C and D) views of the CP. MLVs were located in the dura of the ethmoid bone (lymphatic bed 7, green arrowheads) and around CP foramina (orange arrowheads in B and C). No LYVE1^+^ vessels were found to cross the CP toward the olfactory epithelia (B and E), while the outflow of tracer filled the OE and RE (A and D). Inset in B: Magnification of dotted frame showing tracer-labeled phagocytic cells (yellow arrowheads) concentrated close to the ethmoid MLVs. Ethmoid MLVs (C) are not associated with vWF^+^ blood vessels (D). **(E)** The ethmoid MLVs (white arrowheads) converge dorsally with the lymphatic vessels prolonging the SOS and exit the nasal cavity via the posterior ethmoid foramen (pef) and the foramen cecum (fc). SP, septum. **(F and G)** Lymphatic vessels of the nasal cavity. Sagittal views showing the tracer-labeled LYVE1^−^ vasculature of the OE (F, magenta), the LYVE1^+^ lymphatics of the basal RE and the NP (F, green), and the *Vegfr3*-expressing vessels of the OE (GFP reporter, green in G). **(H)** Sagittal schematic of lymphatic circuits of the nasal cavity. Ethmoid MLVs (LYVE1^+^, light green, 7) do not cross the CP along with olfactory nerves but exit rostrally into the nasal cavity via the pef and fc. OE lymphatics (PROX1^+^/VEGFR3^+^/LYVE1^−^, bluish green) transport CSF, likely collected from perineural drainage along olfactory nerves, then drain into LYVE1^+^ vessels (dark green) of the basal RE and NP that collect into cervical LNs. **(I–K)** Lymphatic drainage from the orbital cavity. Tracer deposits (OVA-A^555^, magenta) and lymphatic vasculature (green in I and J). **(I)** Ventrolateral view of the nasal lacrimal sac region (NLS, dashed line) shows tracer deposits between the orbital cavity under the eye (E) and the olfactory epithelium (OE). Orbital LYVE1^+^ lymphatics connect with facial lymphatics (white arrowheads). **(J)** Lymphatic tracer uptake by orbital PROX1^+^ vessels (white arrowhead). **(K)** Macroscopic imaging of a mouse head 10 min after ICM injection with OVA-A^555^ (magenta) and intra-ocular delivery of OVA-A^488^ (green). Both OVA-A^488^ and OVA-A^555^ collected into the associated mandibular LNs (white); only OVA-A^555^ drained into the mandibular LNs (magenta). **(L–N)** Lymphatic drainage from the nose. **(L)** Coronal view of the nose showing two drainage circuits: dorsal (white arrowheads) and ventral (yellow arrowheads). Dashed circles show the distal part of NLD with LYVE1^+^ LVs (green) costained with OVA-A^555^ (magenta). **(M)** Macroscopic imaging of mandibular LNs (mLN, green arrowhead) labeled with OVA-A^488^ 5 min after nostril OVA^488^ injection. **(N)** Sagittal schematic of lymphatic drainage circuits from the orbital cavity and the nose toward cervical LNs. A, anterior; D, dorsal; L, lateral; P, posterior; PEF, posterior ethmoid foramen; V, ventral. Scale bar: 180 μm (insert in B); 500 μm (A–C, E–J, and L); 80 μm (D); 2 mm (K and M).

**Figure S3.**
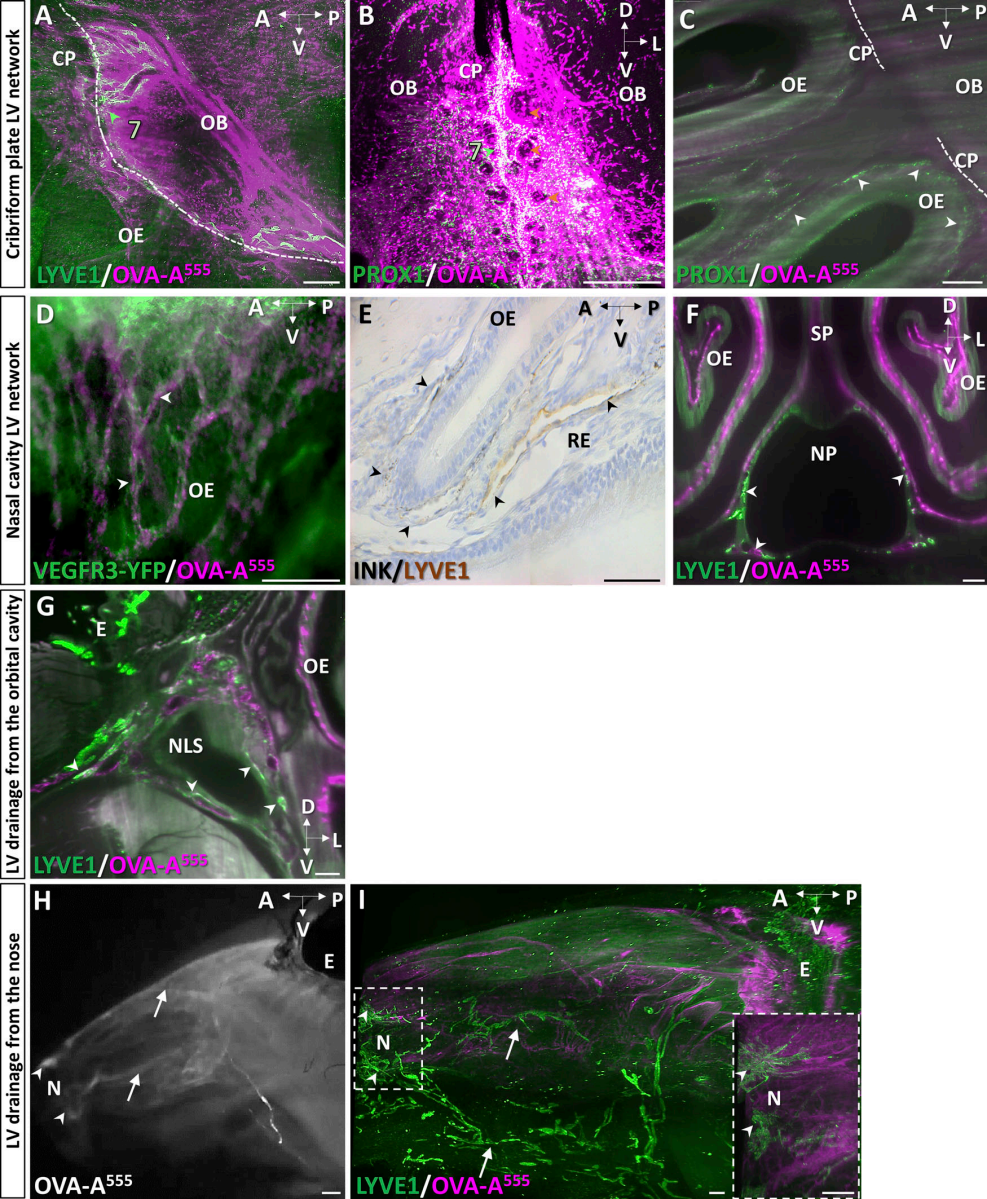
**CSF drainage through the cribriform plate and inside the nasal cavity. (A–F)** Pattern of tracer 45 min after ICM (A) or Th-Lb (B–F) injections of OVA-A^555^ (magenta) or ink. **(A and B)** Sagittal (A) and coronal (B) views of the ethmoid MLVs (7). Note tracer deposits at the cribriform plate (CP) border and in LYVE1^+^ (A, green arrowhead) and PROX1^+^ (B, green arrowhead) MLVs around olfactory nerve foramina (orange arrowheads). Dotted line, limit between intra- and extracranial regions; OB, olfactory bulb; OE, olfactory epithelium. **(C–F)** Sagittal (C–E) and coronal (F) views of nasal cavity lymphatics. Tracer deposits (magenta in C, D, and F; ink in E) are colocalized with lymphatic markers: PROX1 (C, green), VEGFR3-YFP (D, green), and LYVE1 (E and F). **(C)** PROX1^+^ lymphatics in the turbinate, on the outer aspect of the CP (white arrows in C). **(D)** VEGFR3^+^ lymphatics transporting OVA-A^555^ tracer in the OE (white arrowheads). **(E)** Two contiguous paraffin sections of the nasal cavity showing ink tracer (black arrowheads) inside narrow LYVE1^−^ vessels and collecting LYVE1^+^ lymphatics in the respiratory epithelium (RE). **(F)** LYVE1^+^ lymphatics, including tracer-labeled vessels around the nasopharynx (NP). SP, septum. **(G)** Lymphatic drainage from the orbital cavity. Coronal view showing tracer deposits (magenta) and pattern of LYVE1^+^ lymphatics (green). Lymphatic-tracer uptake is detected around the nasal lacrimal sac (NLS, white labeling, arrowheads). E, eye. **(H and I)** Lymphatic drainage from the nose 90 min after intraspinal OVA-A^555^ injection (magenta). Macroscopic (H) or LSFM (I) imaging showing tracer accumulation at the level of the nostril (white arrowheads in H and I) and tracer drainage along dorsal and ventral nasal veins (white arrows in H). Note LYVE1^+^ lymphatics in the nostril (arrowheads in the magnified frame in I) and along nasal veins (arrows in I). Scale bar: 300 μm (A, H, and I, insert in I); 150 μm (B–F).

The lamina cribrosa of the ethmoid bone, also called the cribriform plate, is a main CSF outflow pathway from the mouse skull (Norwood et al., 2019). Whether MLVs extend through the cribriform plate toward the nasal cavity and contribute to this drainage pathway remains unclear (Proulx, 2021). As shown on a lateral view of the forebrain and the nasal cavity (Fig. 5 A), intraspinally injected OVA-A^555^ remained concentrated along perivascular spaces of the cortex and olfactory bulbs, at the cribriform plate, and downward in the olfactory and respiratory epithelia. Between the olfactory bulbs, the ethmoid bone showed LYVE1^+^ MLVs that surrounded olfactory nerve foramina on the dural side of the cribriform plate (Fig. 5, B and C). Ethmoid MLVs located near dense populations of OVA-A^555+^ phagocytic cells and contained OVA-A^555^ in LYVE1^−^ and PROX1-expressing vessels (Fig. 5 B and Fig. S3, A and B). Unlike other MLVs, ethmoid MLVs were not associated with dural vWF^+^ veins or sinuses (Fig. 5 D). We failed to detect extension of ethmoid MLVs across the ventral and central part of the cribriform plate toward the nasal cavity (Fig. 5 B and Video 7). However, in the most dorsorostral part of the cribriform plate, a discrete connection was observed between ethmoid MLVs and the superior nasal cavity via the foramen cecum and the posterior ethmoid foramen (Fig. 5, B and E). Ethmoid MLVs thus connect dorsally with the rostral extension of the MLVs of the superior olfactory sinus, and ventrally with cavernous MLVs. They may have only a limited contribution to nasal CSF outflow through the cribriform plate.

**Video 7.** **LYVE1**^**+**^ **MLVs localized around the olfactory bulbs.** No LYVE1^+^ lymphatic connection crossed the cribriform foramina via the nasal cavity, which harbors a dense network of OVA-A^555+^ vessel-like structures. Frame rate, 24 frames/s.

In the superior and middle part of the nasal cavity, downstream of the cribriform plate, the absence of LYVE1^+^ lymphatics (Fig. 5 F) contrasted with the presence of a dense network of OVA-A^555^–positive capillaries that expressed VEGFR3 and PROX1 (Fig. 5 G and Fig. S3, C and D). LYVE1 expression reappeared in tracer-labeled vessels in the basal domain of the respiratory epithelium and along the nasopharynx (Fig. 5 F and Fig. S3, E and F), which likely represent collector lymphatics downstream of the VEGFR3^+^/PROX1^+^/LYVE1^−^ vessels in the upper nasal region. The nasopharynx provided a major route for lymphatics draining OVA-A^555^ rostrocaudally toward cervical LNs (Fig. 5 F). Altogether, these observations indicate that lymphatics of the upper nasal cavity are distinct from MLVs, without physical continuity and phenotype similarity, although both lymphatic circuits contribute to CSF drainage (Fig. 5 H).

Finally, we focused on the pathways of OVA-A^555^ outflow and lymphatic drainage from the orbital and nasal cavities. In the ocular region, we found LYVE1^−^ and PROX1-expressing LVs with OVA-A^555^ deposits in the nasolacrimal sac area (Fig. 5, I and J; and Fig. S3 G). Nasolacrimal LYVE1^+^ LVs directly collected into the associated-mandibular LNs. As shown in Fig. 5 K, a periorbital delivery of OVA-A^488^ (green) after ICM injection of OVA-A^555^ (magenta) led to the drainage of green tracer exclusively into the associated-mandibular LNs (white), and not into mandibular LNs (magenta). In the nose, we identified two foci and drainage circuits (white arrowheads in Fig. 5, A and L). LYVE1^+^ LVs containing OVA-A^555^ deposits were detected in the basal respiratory epithelium and around the distal part of the nasolacrimal duct (yellow arrowheads in Fig. 5 L). The anterior nasal cavity drained directly into mandibular LNs as demonstrated by the labeling of these nodes 5 min after nostril OVA^488^ injection (Fig. 5 M and Fig. S3, H and I).

Therefore, intraorbital LVs drain CSF/ISF from the CAV, while nasal LVs collect the CSF outflow from the cribriform plate. These orbital and nasal lymphatic circuits separately collect into the associated-mandibular and mandibular LNs, respectively (Fig. 5 N).

### Caudal lymphatic drainage of CSF toward vertebral LNs

**Figure S4.**
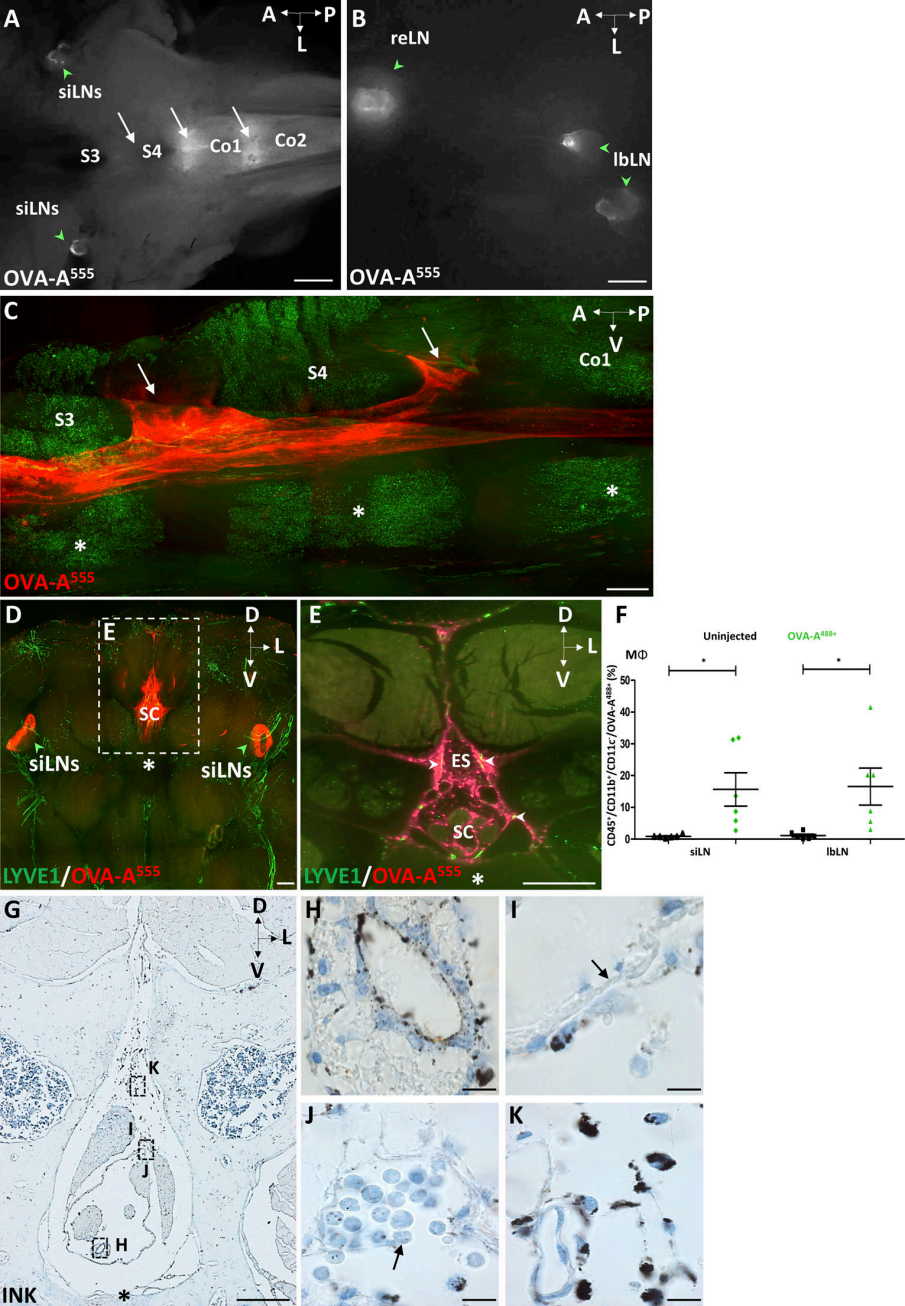
**Sacral spinal cord outflow. (A and B)** Macroscope imaging of the sacrococcygeal region 90 min after ICM injection of OVA-A^555^ tracer. **(A)** Tracer deposits were detected at intervertebral spaces (white arrows, S3–S4, S4-Co1, Co1–Co2) and in the sciatic LNs (siLN, green arrowheads). **(B)** In the peritoneal cavity, the tracer (white) was detected in collecting lumbar LNs (lbLN) and renal LNs (reLN) (green arrowheads). Co, coccygeal vertebrae; S, sacral vertebrae. **(C–E)** LSFM sagittal (C) and coronal (D and E) views of clarified sacral vertebrae, spinal cord, and dura mater. **(C)** Outflow of tracer (red) in the epidural space (white arrows) between S3–S4 and S4-Co1. Asterisks, vertebral bodies. **(D and E)** Pattern of LYVE1^+^ lymphatics (green) and tracer (red) in the S3–S4 region. Tracer deposits (red) were detected in the vertebral canal and in siLNs (green arrowheads in D). **(E)** Magnification of dotted frame in D. Tracer accumulated in the epidural space (ES), including in LYVE1^+^ lymphatics (yellow, white arrowheads in E). A, anterior; D, dorsal; L, lateral; P, posterior; V, ventral. **(F)** Quantifications of OVA-A^555+^ MΦ (CD45^+^/CD11b^+^/CD11c^−^) among total CD45^+^ cells FACS-sorted from the siLNs and lbLNs of noninjected mice or mice injected into the Th-Lb spine with OVA-A^488^. *n* = 6 mice/group. Data show mean + SEM; one-way ANOVA with Dunn’s multiple-comparisons test; *, P < 0.05. **(G–K)** Light-field microscope images of coronal paraffin sections of S3–S4 vertebrae from mice with intraspinal injection of ink tracer. **(G)** Low magnification of highly magnified areas shown in H–K. Tracer was detected on the ependymal layer and the pial surface of the spine (H), as well as inside phagocytic cells of the subarachnoid (I and J) and epidural (K) spaces. Note the thin monolayer of dura mater (black arrow in I) and the accumulation of tracer-containing mononuclear cells in subdural arachnoid sacs (black arrow in J). Asterisk, vertebral body. Scale bar: 2 mm (A and B); 500 μm (C–E and G); 10 μm (H–K).

Clearance of CSF has also been shown to occur at the caudal end of the spine via lymphatic vessels collecting into sacral and iliac LNs (Ma et al., 2019). To examine the caudal outflow of CSF in the sacral region of the spinal cord, we performed ICM injection of OVA-A^555^ and sacrificed mice 90 min later. Under a binocular microscope, OVA-A^555^ deposits were observed in the intravertebral spaces of the coccygeal and sacral vertebral column, between S3 and Co2, as well as in the sciatic, lumbar and renal LNs (Fig. S4, A and B). LSFM imaging confirmed that OVA-A^555^ accumulated in the dural and subarachnoid space all along the spinal cord, and specifically in the epidural space between S3 and Co2 (Fig. S4, C–E). Tracer-labeled MΦ (CD45^+^/CD11b^+^/CD11c^−^) were found in sciatic and lumbar LNs by flow cytometry analysis after Th-Lb injection of OVA-A^488^ (Fig. S4 F). Finally, a thinning in the dorsal dura mater layer and the presence of clusters of ink-labeled phagocytic cells were detected in the epidural S3–Co2 region on paraffin sections of sacral vertebral segments isolated after ICM injection of ink (Fig. S4, G–K). These data confirm sacral CSF outflow (Ma et al., 2019) and indicate that epidural myeloid cells uptake CSF antigens in the sacral spine and LNs.

### 3D mapping of the dural vasculature in humans

**Figure 6.**
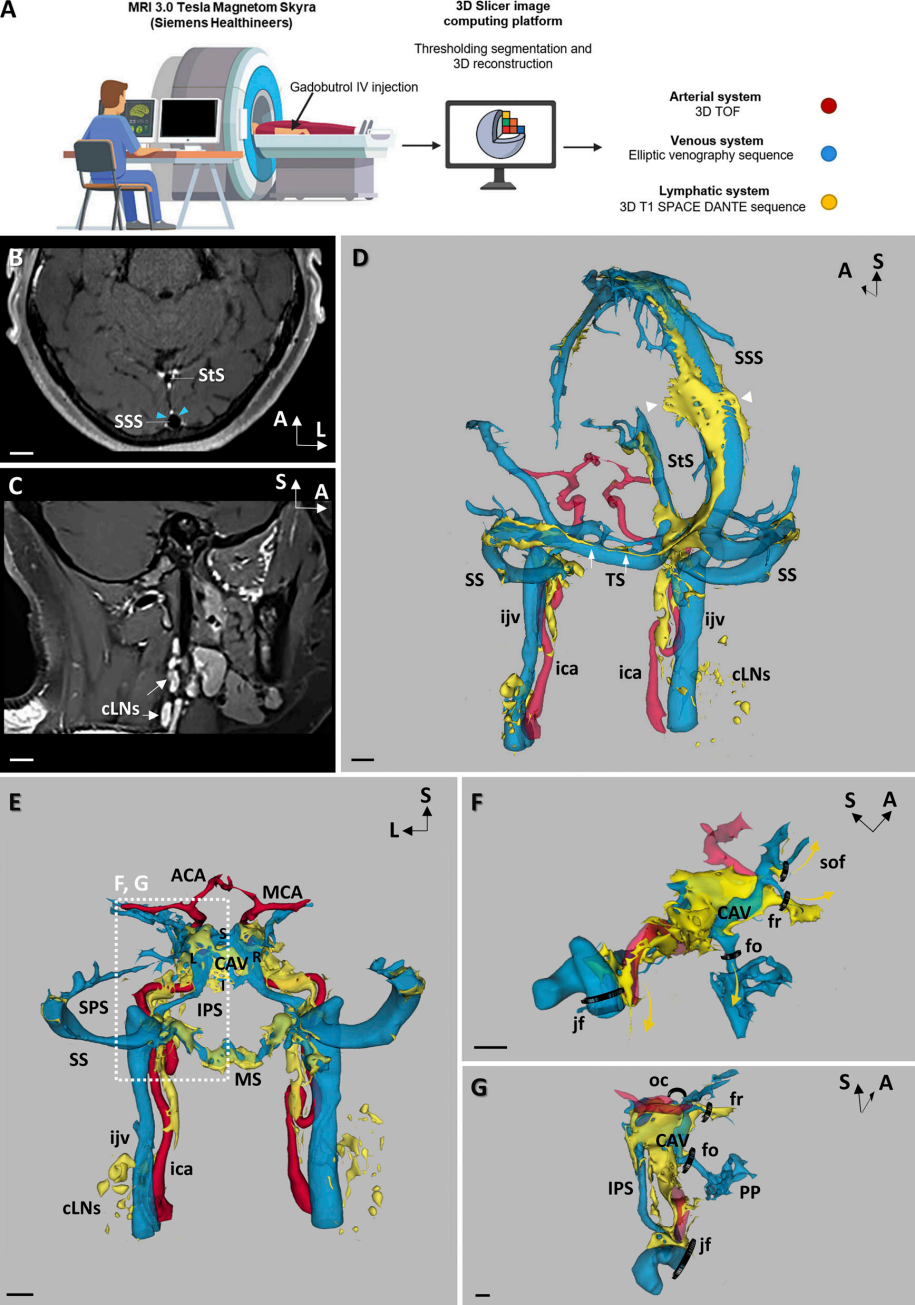
**Meningeal vascular MRI in humans. (A)** Schematic of the workflow for meningeal vascular 3D mapping by MRI in humans. Native sequences were acquired from a 3T MRI before and after i.v. gadobutrol injection. 3D-Slicer platform was used for semiautomated signal intensity–based thresholding and segmentation of native sequences. **(B–G)** Native VW-MRI (B and C) and meningeal vascular 3D mapping (D–G) after gadobutrol injection of a patient with MS (number 7 in Table 1). **(B)** Native 3D T1 SPACE DANTE in an oblique axial plane crossing the transverse axis of both the superior longitudinal (SSS) and the straight sinuses (StS). The black-blood contrast allowed to darken the lumen of venous sinuses, while dural lymphatics (white) were strongly enhanced by gadobutrol and precisely segregated from the unenhanced perisinusal dura mater (blue arrowheads). **(C)** Native 3D T1 SPACE DANTE sequence in a sagittal plane along the longitudinal axis of the left internal jugular vein (ijv) covering both the head and neck. cLNs (arrows) surrounding the ijv were enhanced by the contrast agent and distinct from adjacent soft tissues. **(D)** Oblique posterior view of the dorsolateral group of dural venous sinuses (blue), including the SSS, the StS, the transverse (TS), and the sigmoid sinuses (SS). The ijv represents the major venous outflow of the dorsolateral group of sinuses. Dorsolateral perisinusal fluids (yellow) concentrate in perisinusal areas and include vessel-like compartments (arrows) associated with flattened vesicles (arrowheads). Perisinusal fluids were detected along the internal carotid arteries (ica) and until the dcLNs. **(E)** Posterior view of the meningeal vascularization in the anterior part of the skull. Left (L) and right (R) CAV are connected at the midline by the superior (S) and inferior (I) coronary sinuses. The superior petrosal sinus (SPS) connects the CAV with the SS, while the inferior petrosal sinus (IPS) connects the CAV with the IJV. The IPS is also connected with the marginal sinus (MS), which drains caudally in the perivertebral venous plexuses. In the intracavernous segments, the ica crosses the CAV before intradural bifurcation in middle (MCA) and anterior (ACA) cerebral arteries. Perisinusal fluids were detected in the perisinusal areas of the CAV, the MS surrounding the foramen magnum, and along the IPS. Exit routes of perisinusal fluids from the skull followed the pericarotid route in the carotid canal, anteriorly, and the perivertebral canal along the vertebral arteries, posteriorly. **(F and G)** Parasagittal (F) and posterior (G) views of fluid exit routes from the CAV perisinusal area, showing the pericarotid route inside the carotid canal as well as several transforaminal routes along the branches of trigeminal nerve, including the superior orbital fissure (sof) along the ophthalmic branch, the foramen rotundum (fr) along the maxillary branch, and the foramen ovale (fo) along the mandibular branch. Besides their specific trigeminal nerve branches, these foramina contain corresponding emissary veins that collect extracranially in the extracranial veins, including the pterygoid plexuses (PP). No perisinusal flow was observed through the optical canal (oc). Scale bar: 1 cm (B–G).

To explore the organization of the human dural lymphatic vasculature, we enrolled patients with neurovascular or neurological diseases that required contrast-enhanced MRI, including patients with idiopathic intracranial hypertension (IIH; *n* = 5), multiple sclerosis (*n* = 4), unilateral jugular stenosis (JS; *n* = 1), and Gorham–Stout disease (GSD; *n* = 1) (Table 1). The patients received a systemic injection of gadobutrol, and the scans were processed using 3D-image reconstruction software (Fig. 6 A). The flow of the contrast agent was imaged sequentially, first in the blood vessels, and then after it reached the lymphatic vasculature. We used an MR elliptic venography sequence after gadobutrol injection, followed by a 3D T1 SPACE (variable flip angle turbo spin echo) VW imaging sequence modified by addition of a DANTE (delay alternating with nutation for tailored excitation) module (Siemens, Healthineers). Acquisition of the T1 SPACE DANTE sequence was performed ≥15 min after gadobutrol injection. The T1 SPACE DANTE sequence allowed to accurately segregate the slow-flow circuits of the lymphatic vessels from the faster flow circuits of arteries, veins, venules, and CSF in a 6-min scanning time. The combination of the elliptic venography and T1 SPACE DANTE sequences resulted in large field and submillimeter-resolution images of the blood and lymphatic vasculature in the meninges and the neck regions (Fig. 6, B and C). We then generated a 3D map of the different gadobutrol flow circuits from the native sequences. As shown in Fig. 6 D, the slower-flow circuit (yellow) was concentrated in the perisinusal areas along the superior sagittal, straight, transverse, sigmoid, and CAV, standing apart from venous sinuses and veins (blue), and included vessel-like compartments (arrows) associated with flattened vesicles (arrowheads). In the neck region, extracranial lymphatics connected dural lymphatic vessels with cervical LNs (Fig. 6 D).

**Table 1.** Clinical features and volumetric quantifications of the study patients

Patient	Age (yr)	Gender	Neurological disease	Medication	TIV (mm^3^)	Normalized MLV volume	Normalized venous volume
1	64	F	IIH + CSF leak		1,414,960	3,770.45	19,190.93
2	73	F	IIH + CSF leak		1,397,400	3,429.09	13,044.44
3	49	F	IIH	Acetazolamide	1,158,840	2,474.28	10,802.18
4	40	F	IIH	Acetazolamide	1,273,760	2,579.78	13,746.00
5	25	F	IIH	Acetazolamide	1,270,220	2,479.75	14,631.01
6	42	M	JS		1,432,380	7,500.31	10,156.52
7	27	M	MS with acute demyelinating event	Tecfidera 480 mg; methylprednisolone (3 g) <10 d	1,546,820	7,372.09	13,081.61
8	38	F	MS with acute demyelinating event	Methylprednisolone (3 g) <10 d	1,094,560	2,786.73	10,692.70
9	54	F	MS		1,312,850	3,142.83	16,197.20
10	67	M	MS	Dimethyl fumarate (14 mg)	1,464,580	7,703.16	22,947.60
11	37	M	GSD		1,382,580	15,470.42	20,718.80
Mean (SEM)	47 (4.82)	4:7 (M:F)			1,340,814 (40,519.6)	5,337.17 (1,200.30)	15,019.00 (1,292.32)

F, female; M, male. The volumes of MLVs and venous sinuses were normalized against the TIV.

**Figure S5.**
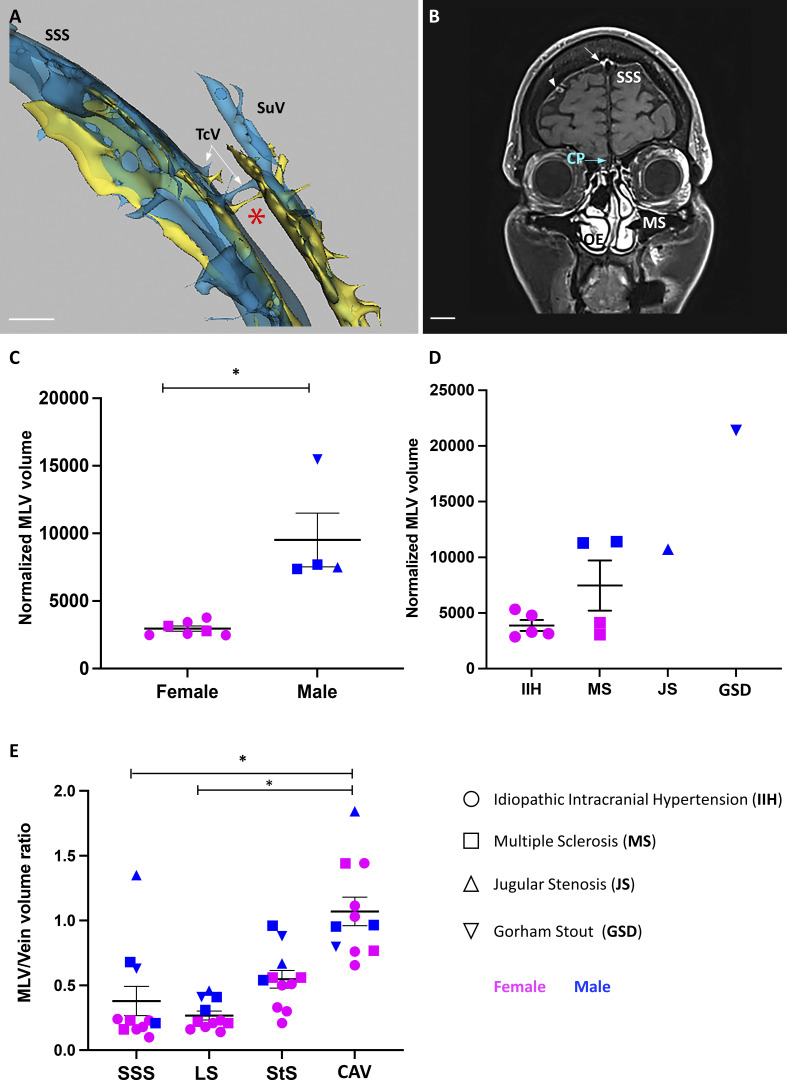
**Meningeal and skull vascular MRI in humans. (A and B)** VW-MRI (A) and meningeal vascular 3D mapping by MRI (B) of a patient with IIH and CSF leak (patient 1 in Table 1). **(A)** Transcalvarial connections at the level of the superior longitudinal sinus (SSS). A few transcalvarial veins (TcV) connecting the SSS with subcutaneous veins (SuV) and the systemic circulation were associated with tiny nonvenous channels filled with perisinusal fluids (red asterisk). **(B)** Native 3D T1 SPACE DANTE sequence. Coronal imaging plane across the cribriform plate (CP) and the maxillary sinuses (MS). Intracranially, enhanced gadobutrol signal was detected along the SSS (white arrow) and the perivenous space of several cortical veins (white arrowhead). Extracranially, the olfactory epithelium (OE) was strongly enhanced after gadobutrol injection, but no gadobutrol signal was observed from the dura mater across the CP (blue arrow). Scale bar: 1 cm (A and B). **(C–E)** Quantitative MRI of human MLVs. Data show mean + SEM; one-way ANOVA with Dunn’s multiple-comparisons test; *, P < 0.05. **(C and D)** MLV volume measurements between females and males (C) and between neurological disorders (D). For each patient, MLV volume was normalized against the TIV. MLV volume was greater in males (*n* = 4) than in females (*n* = 7). No significant difference was observed between groups of patients, except for the male patient with GSD, who showed the greatest MLV volume. **(E)** MLV/vein volume ratio in the superior sagittal (SSS), the straight (StS), and the lateral (LS) and cavernous (CAV) sinuses. MLVs of CAV have a higher MLV/vein volume ratio compared with other MLV beds and show higher interindividual variation. Data show mean + SEM; one-way ANOVA with Dunn’s multiple-comparisons test; *, P < 0.05.

Interestingly, parallel to the superior sagittal sinus, we found perforating venules and slow-flow channels crossing the skull and connecting to superficial intracalvaria and subcutaneous lakes (Fig. S5 A). Connection of dural channels with calvaria bones has been previously observed in the mouse (Cai et al., 2019; Pulous et al., 2021 *Preprint*), and recently in humans (Ringstad and Eide, 2021). In the anterior region of the skull, gadobutrol slow-flow was detected in the perisinusal area of the CAV (Fig. 6, E–G). In the inferior region of the skull, the marginal sinus around the foramen magnum was also surrounded by gadobutrol slow-flow that extended caudally along the vertebral arteries (Fig. 6 E). Like dural lymphatics in the mouse, the regional gadobutrol slow-flow circuits were interconnected between the CAV and the jugular vein and between the marginal sinus and the jugular vein (Fig. 6, E–G).

The exit of lymphatics from the skull was observed along blood vessels through the jugular foramen for dorsolateral lymphatics and along the carotid canal, the superior orbital fissure, the foramen rotundum, and the foramen ovale for lymphatics of the CAV (Fig. 6, F and G). In the most frontal region of the skull, we failed to detect lymphatic connections with the nasal conchae through the central part of the cribriform plate (Fig. S5 B).

MLV quantifications were performed for each of the 11 patients by two independent neuroradiologists, with a 0.9–1 intraclass correlation coefficient (ICC) that demonstrated reproducible quantification. We found that MLV volume was significantly different between genders (Fig. S5 C), but not between neurological disorders except for GSD (Fig. S5 D). The MLV/vein volume ratio was higher for the CAV, with a large interindividual variability, and lower for the straight, lateral, and superior sagittal sinuses (Fig. S5 E).

### MLV hypertrophy in a patient with GSD

**Figure 7.**
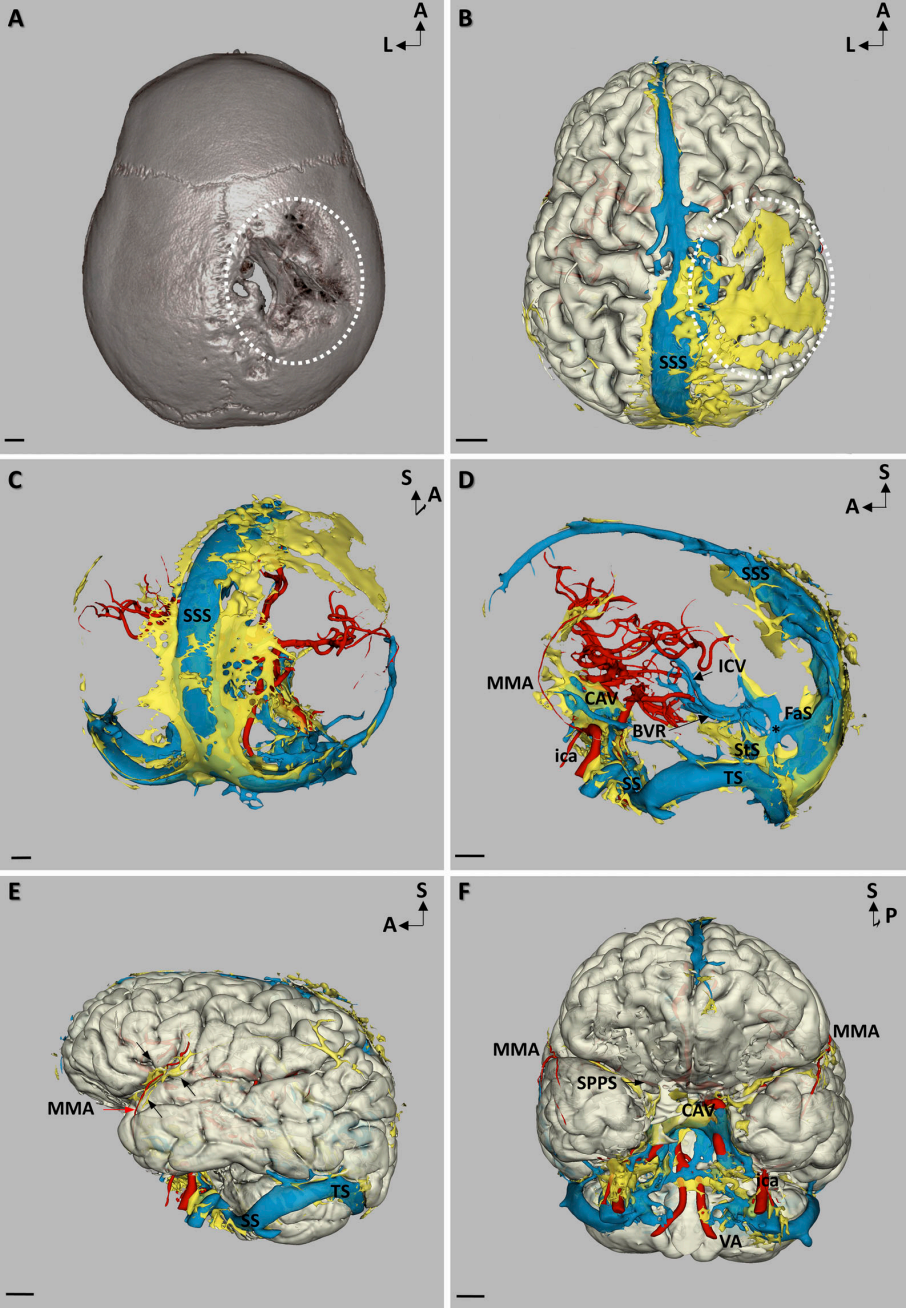
**Altered MLV imaging overlaps with skull bone erosion in a patient with GSD. (A)** 3D reconstruction of a computed tomography scan in superior view showing the large erosion of the right parietal bone (white circle). **(B–F)** Meningeal vascular 3D mapping by MRI. **(B)** Superior view showing a large area of gadobutrol slow-flow (yellow) in the region of the vanished bone (white circle) and extending to the perisinusal area of the SSS. **(C)** Posterior view showing the extension of the perisinusal gadobutrol enhancement (yellow) all along the SSS. **(D)** Lateral view showing that MLV hypertrophy involved MLVs of the SSS, StS, LS, SS, and CAV. This MLV hypertrophy was associated with a malformation of the StS that was replaced by a large venous plexus (asterisk) encompassing the StS and an abnormally persistent embryonic falcine sinus (FaS). **(E)** Lateral view reconstruction highlighting the close relationships between the MMA (red arrow) and its related MLVs (black arrows). **(F)** Anterior view allowing the visualization of the CAV, ica, and vertebral arteries (VA). MLV hypertrophy allowed to visualize the continuity of MMA-related MLVs toward the skull base. Note that, instead of lining the MMA toward the spinosum foramen, MLVs followed the middle meningeal vein toward the sphenoparietal sinus (SPPS) toward the CAV. Scale bar: 1 cm (A–F).

The patient with GSD showed extensive MLV hypertrophy compared with the other patients (Fig. S5, C–E). GSD, also known as vanishing bone disease, is a rare disease with ∼300 cases reported since its initial description (Gorham and Stout, 1955; Dellinger et al., 2014). Two cases were associated with oncogenic KRAS mutations in affected lymphatics (Homayun-Sepehr et al., 2021); the mutational status of the patient we examined is yet unknown. GSD is characterized by ectopic lymphatic vessel proliferation in bones, resulting in progressive osteolysis (Fig. 7 A). A strong and diffuse enhancement of slow-flow gadobutrol was observed in the vanished parietal bone and the associated dural area of this patient (Fig. 7 B). The increased gadobutrol signal on T1 SPACE DANTE sequence was not restricted to the right parietal dura but was also observed along the superior sagittal sinus (Fig. 7 C) as well as along the middle meningeal arteries (MMAs) and the CAV (Fig. 7, D–F). MLVs adjacent to the MMA were not detected in the other patients of our study but were identified in mice (Antila et al., 2017; Ahn et al., 2019).

## Discussion

**Figure 8.**
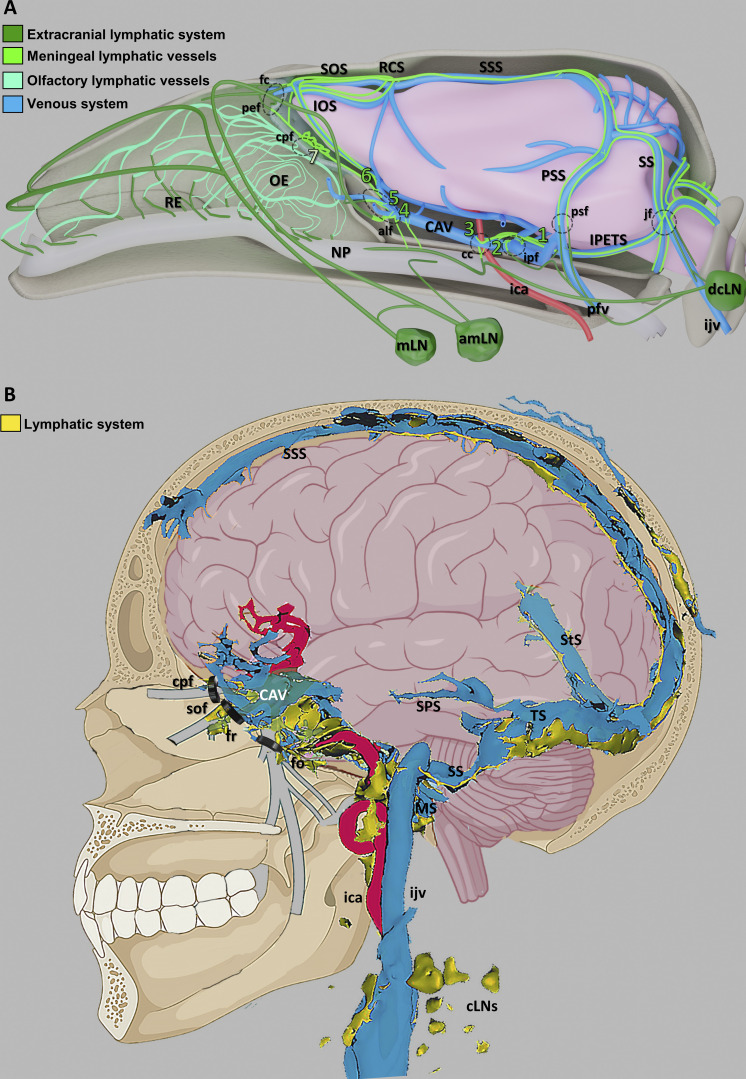
**Schematic representation of the dural venolymphatic complex in mice and humans. (A)** Summary schematic of lymphatic CSF drainage circuits in the mouse head. Cerebral veins and dural sinuses (blue) drain blood from the brain. 1–6, novel MLV uptake sites where perivenous glymphatic efflux from these regions communicates with perisinusal areas of the dura mater. Fluorescent green, MLVs; bluish green, olfactory lymphatic vessels; dark green, extracranial lymphatic system; red, internal carotid artery; beige, nasopharynx; amLN, accessory mandibular LN; cc, carotid canal; cpf, cribriform plate foramina; fc, foramen caecum; ica, internal carotid artery; ijv, internal jugular vein; IOS, inferior olfactory sinus; IPETS, inferior petrosal sinus; ipf, interpterygoid foramen; jf, jugular foramen; mLN, mandibular LN; NP, nasopharynx; OE, olfactory epithelium; pef, posterior ethmoid foramen; pfv, posterior facial vein; psf, petrosquamous fissure; PSS, petrosquamous sinus; RCS, rostral confluence of sinuses; RE, respiratory epithelium; SOS, superior olfactory sinus; SS, sigmoid sinus SSS, superior sagittal sinus. **(B)** Schematic representation of the dural venolymphatic complex and lymphatic outflows in humans. Signal enhancement of gadobutrol (yellow) was depicted around most dural sinuses, including the SSS, the straight sinus (StS), the transverse sinus (TS), the sigmoid sinuses (SS), and the CAV. Gadobutrol flow was also detected in the carotid canal along the ica or in transforaminal routes along the trigeminal nerve branches (gray) through the superior orbital fissure (sof), the foramen rotundum (fr), and the foramen ovale (fo). cLN, cervical LNs; ijv, internal jugular vein; MS, marginal sinus.

In this work, we tracked lymphatic drainage in the dura mater using intraspinal/ICM administration of OVA in mice or systemic gadobutrol injection in humans. Remarkably, both approaches resulted in a similar pattern of tracer uptake around dural venous sinuses and revealed draining to cervical LNs via tracer-containing MLVs. Therefore, the dural lymphatic circuits of CSF drainage identified in the mouse mirrored the perisinusal lymphatic drainage circuits mapped by MRI in the human dura mater (Fig. 8, A and B; and Video 8).

**Video 8.** **3D schematic of MLVs and cranial lymphatic circuits collecting into mandibular and cervical LNs in the adult mouse. **Frame rate, 25 frames/s.

The 3D maps of dural lymphatics in mice and humans reveal lymphatic drainage around the CAV that connected with dorsal and basal lymphatics and drained through the foramina and fissures of the skull. These observations extend recent reports on the presence of lymphatics at the exit of trigeminal nerves (Mezey et al., 2021; Albayram et al., 2022) and suggest that cavernous lymphatics specifically drain perivenous efflux from their tributary cerebral veins into collecting scLNs and dcLNs, thereby providing a region-specific drainage of the glymphatic outflow from the dura mater to cervical LNs. The conserved functional anatomy of dural lymphatics between mice and humans underscores that murine models are relevant to predict the pathophysiological contribution of the dural venolymphatic complex and test lymphatic-targeted drugs in neurological disease models.

Our findings underline the critical role of MLVs in the uptake and drainage of the glymphatic efflux from perisinusal and perivenous spaces. In mice, the pattern of lymphatic uptake of tracers was similar after injection into the caudal spine and the cisterna magna, indicating that CSF lymphatic uptake and drainage occurs independently of the tracer injection site. This dural lymphatic uptake may preserve the subarachnoid CSF from pollution by the brain-derived waste transported in the perivascular space of cerebral veins. The perisinusal spaces in the dura mater, moreover, provide an interface for uptake of CSF- and brain-derived antigens by dural phagocytes and presentation to meningeal T cells (Rustenhoven et al., 2021). MLVs are thus conveniently positioned for transferring CNS-derived antigens, CNS-antigen presenting cells, and activated T cells from the dura mater to the collecting LNs. Our data support a model of antigen transport from the perivascular cerebral venous spaces into the dural lymphatics, which involves the newly discovered uptake sites at the CAV. As we trace OVA uptake into MLVs in mice after intraspinal injection over time, we cannot distinguish if the OVA was taken up by phagocytic cells in the meninges that then migrate into the LNs, or if naked OVA reached the LNs and was phagocytosed in antigen presenting cells already present in the LNs. The latter model is consistent with the rapid drainage kinetics of ICM injected OVA-A^555^ and with previous studies demonstrating that dural myeloid cells can migrate into dcLNs, but their number is low and their migration to dcLN occurs over 1–2 d (Louveau et al., 2018).

The lack of robust anatomic continuity between MLVs and the nasal cavity strongly suggests that MLVs have no major contribution to the nasal CSF outflow in the mouse. In humans, the contribution of lymphatics to cribriform plate drainage was also not supported by VW-MRI data. We propose that the ethmoid MLVs (large blind ended LYVE1^+^ capillaries) and the lymphatics of the upper/middle olfactory epithelium (narrow LYVE1^−^ vessels) represent two distinct lymphatic beds, with MLVs draining the glymphatic outflow and the olfactory lymphatics draining the subarachnoid CSF and the nasal mucosa. The expression of VEGFR3 by both ethmoid MLVs and olfactory lymphatics suggest that their development and maintenance are VEGF-C dependent. We previously showed that MLV development occurs postnatally in a VEGF-C–dependent manner, in that neonatal VEGF-C inhibition using various means resulted in the near-complete absence of MLVs and impaired the drainage of intracerebrally injected microspheres into the dcLNs (Antila et al., 2017). Hence it is likely VEGF-C/VEGFR3 signaling also regulates cavernous MLV development in the anterior region of the skull, although this remains to be formally proven.

The initial description of human MLV imaging using VW-MRI techniques and the 3D T1 SPACE sequence (Absinta et al., 2017) has been extended by other MRI protocols based on intrathecal (Eide et al., 2018; Ringstad and Eide, 2020) or intravenous gadobutrol injection (Cao et al., 2020; Park et al., 2020; Wu et al., 2020). Intrathecal injections are poorly suitable for clinical practice due to their invasiveness (Eide et al., 2018; Ringstad and Eide, 2020). 3D T2-FLAIR MRI without contrast media administration (Albayram et al., 2022) is non-invasive and relies on the detection of internal signals from protein-rich lymphatic fluids. However, the T2-FLAIR iso-signal is not selective for MLVs and therefore precludes signal-intensity based thresholding and quantitative MLV analysis. Here we incorporated a DANTE module into the 3D T1 SPACE sequence with gadobutrol to improve the suppression of the residual slow-flow signals, which allowed for a precise and reliable extraction of MLVs using semiautomated signal intensity–based thresholding. In addition, we completed the VW-MRI protocol with a MR venography sequence, allowing us to unambiguously discriminate lymphatics from venous sinuses and veins, and to facilitate postprocessing segmentation and accurate MLV volume quantification.

The T1 SPACE DANTE sequence revealed transcranial vessels that penetrate the skull and join subcutaneous lymphatic and venous vasculature. Similar trans-osseous channels have previously been identified in the mouse calvaria (Cai et al., 2019; Herisson et al., 2018; Rindone et al., 2021), where they participate in CSF-mediated immune cell trafficking between the calvarial bone and the dura mater and regulate myelopoiesis and egression of myeloid cells into the meninges after a spine injury (Mazzitelli et al., 2022). The MRI surveillance of calvarial channels may thus inform about alterations of CNS immune responses involving the skull bone marrow in patients with neurological diseases or meningeal infections.

The T1 SPACE DANTE sequence allowed a comparative quantitative MRI analysis of MLV volume between individuals with different neuropathological conditions. Only a patient with GSD showed significantly altered MLVs, with a marked hypertrophy of dorsal and latero-basal MLVs in the eroded region of the skull. We speculate that gadobutrol-enhanced VW-MRI will allow longitudinal imaging of disease progression that may be relevant for diagnostic and prognostic imaging. No significant variability of MLV volume was observed between other patients with either IIH, MS, or JS, but patient numbers in each group were low, and additional MRI imaging of subjects without vascular stenosis or neurological diseases is needed to establish if MLV structural features are altered in these CNS disorders.

MLVs have been found to vary with gender in humans (Park et al., 2020) but not in mice (Louveau et al., 2015), and MLV drainage decreased with aging in mice and humans (Da Mesquita et al., 2018; Ahn et al., 2019; Albayram et al., 2022). Sex and age are also known to affect the human immune response (Klein and Flanagan, 2016). The small number of patients we imaged showed no correlation between age and MLV volume (Table 1), but males showed a significantly greater MLV volume compared with females. While the mechanisms by which sex may regulate MLVs and neuroimmunity remain unknown, it is interesting to speculate that the reduced MLV volume in females may relate to their increased vulnerability to IIH, MS, and meningioma.

## Material and methods

### Study approval

All in vivo procedures used in this study complied with all relevant ethical regulations for animal testing and research, in accordance with the European Community for experimental animal use guidelines (L358-86/609EEC). The study received ethics approval by the Ethical Committee of Institut National de la Santé et de la Recherche Médicale (no. 2020071714182580) and the Institutional Animal Care and Use Committee of Institut du Cerveau et de la Moelle épinière.

### Human patients

Procedures in humans have been approved by our institutional review board (#CRM-2111-216; Comité d’Ethique pour la Recherche en Imagerie Médicale). After informed written consent, we retrospectively collected the clinical and radiological data of 11 patients who underwent MRI with gadobutrol injection according to the protocol described below. Five of them were explored for IIH, four for MS, one for unilateral JS, and one for a GSD. Radiological data were anonymized, and postprocessing was performed by two experienced neuroradiologists.

### Animals

Male and female C57BL/6J mice, *Vegfr3YFP* (Calvo et al., 2011), or *Vegfr3-CreERT2*; *mTmG* (unpublished, provided by Prof. Jason Butler, Hackensack University Medical Center, NJ) mice 2–4 mo of age were used for all experiments.

### Intra-CSF injections of tracers in mice

ICM, thoracic-lumbar, and lumbar-sacral injections were performed in adult male and female C57BL/6J and *Vegfr3YFP* mice 8–10 wk of age. Mice were injected i.p. with Buprecare solution and anesthetized by Isoflurane gas (2–3%). Mice were maintained at the head or vertebral level of injection with a stereotaxic apparatus (Stoelting). The skin was incised at neck level, Th12-L1 (thoracic-lumbar injection) or L6-S1 (lumbar-sacral injection) vertebral levels. Muscles were moved to the side until the dura mater was exposed. Meninges were incised using a 30-gauge needle. 2 or 8 μl of OVA-A^555^ (2 mg/ml; Alexa Fluor 555 Conjugate; O34782; Invitrogen) were injected through a microcapillary (Glass Capillaries; GC120-15; Harvard Apparatus) connected to a Hamilton syringe (10 μl). The microcapillary was introduced into one side of the spinal cord parenchyma or above the dura mater at the cisterna magna level. To avoid the release of OVA-A^555^ during the injection, surgical glue was added to close the incision around the glass capillary. Injections were performed slowly (1 μl/min). Once injection was finished, the capillary was maintained for 2 min before retraction, and surgical glue was added to close the hole made by the capillary. Some tracer leak occasionally occurred despite these precautions, leading us to examine tracer drainage at a site distal to the injection site: i.e., cranial drainage was examined in mice after Th-Lb or Lb-Sc injection, while sacral drainage was injected after ICM injection. Tissue incisions were closed with Michel Suture Clips (7.5 × 1.75 mm; 12040-01; Fine Science Tool). After 15, 45, or 90 min, mice were euthanized and perfused as described. To study fluid drainage from the orbital cavity and the nostril, OVA-A^555^ was injected into the periorbital space (5 μl) or under the skin at the tip of the nostril (2 μl), using a BD Micro-Fine syringe (0.3 ml; 783652 3; BD. Tracer drainage into scLNs was examined 10 or 5 min, respectively, after injection.

### Tissue preparation and decalcification

Mice were given a lethal dose of sodium pentobarbital (Euthasol Vet) and perfusion-fixed through the left ventricle with 10 ml ice-cold PBS and 20 ml of 4% paraformaldehyde (PFA) in PBS. To dissect the head and the vertebrae, the skin was completely removed, all the organs were discarded, and the ribs were removed to keep only the vertebral column from the cervical part until the lumbar part with the spinal cord inside. All the surrounding tissues including muscles, eyes, salivary glands, and ligaments were maintained around the skull and the vertebral column. All samples were decalcified for 3 wk in 10% EDTA in 4% PFA/PBS. The head was cut with a microtome blade along coronal, horizontal, or sagittal axes into either three pieces corresponding to the cribriform plate, CAV, and jugular foramen regions or two pieces corresponding to either the dorsal-versus ventral or left-versus-right halves of the head. The spine was cut along the coronal or sagittal axis into pieces ∼0.8 cm thick (two to four vertebrae) corresponding to the cervical and the sciatic regions. The different sample segments were immediately immersed in ice-cold 4% PFA, fixed overnight at 4°C, washed in PBS, and processed for staining.

### Sample pretreatment for iDISCO^+^

We used the immunolabeling-enabled 3D imaging of solvent-cleared organs protocol (iDISCO^+^, http://www.idisco.info; Renier et al., 2014). The steadily increasing methanol concentrations result in modest tissue shrinkage (∼10%), and the transparency of tissues, such as the adult mouse brain, is increased. In detail, fixed samples were dehydrated progressively in methanol/PBS, 20, 40, 60, 80, and 100% for 1 h each with gentle agitation. They were then incubated overnight in a solution of methanol 33%/dichloromethane 66% (DCM; 270997-12 × 100 ml; Sigma-Aldrich). After 2 × 1 h washes with 100% methanol, samples were bleached with 5% H_2_O_2_ in methanol (1 vol 30% H_2_O_2_/5 vol methanol) at 4°C overnight. After bleaching, samples were rehydrated in methanol for 1 h each, at 80, 60, 40, 20%, and PBS with gentle agitation. Samples were washed rapidly with PBS then incubated 2× 1 h in PTx2 (PBS and 0.2% Triton X-100). At this step, they were processed for immunostaining.

### iDISCO^+^ immunolabeling protocol

Pretreated samples were incubated in 20-ml glass bottles (DWK986546; Merck) in PBS/0.2% Triton X-100/20% DMSO/0.3 M glycine at 37°C for 24 h, and then blocked in PBS/0.2% Triton X-100/10% DMSO/6% donkey serum at 37°C for 24 h. Samples were incubated in primary antibody dilutions in PTwH (PBS and 0.2% Tween-20 with 10 mg/ml heparin)/5% DMSO/3% donkey serum at 37°C for 21 d. Samples were washed five times in PTwH until the next day and then incubated in secondary antibody dilutions in PTwH/3% donkey serum at 37°C for 14 d. Samples were finally washed in PTwH five times until the next day before clearing and imaging. We used the following primary antibodies: goat anti-mouse CD31 (1:1,000, AF3628; R&D Systems), chicken anti-GFP (1:2,000, GFP10-20; AVES), goat anti-mouse PDLX (1:1,000, AF1556; R&D Systems), rabbit anti-mouse LYVE1 (1:800, 11-034; AngioBio), goat anti-human PROX1 (1:1,000, AF2727; R&D Systems), rabbit anti-mouse TUJ1 (1:2,000, 802001; BioLegend), and rabbit anti-human vWF (1:300, A0082; Agilent). Primary antibodies were detected with the corresponding Alexa Fluor 555–, 647–, or 790–conjugated secondary antibodies from Jackson ImmunoResearch at 1/1,000 dilution.

### iDISCO^+^ tissue clearing

After immunolabeling, samples were dehydrated progressively in methanol in PBS, 20, 40, 60, 80, and 100% each for 1 h with gentle agitation. They were then incubated overnight in a solution of methanol 33%/DCM 66% followed by incubation in 100% DCM for 2× 1 h to wash the methanol. Finally, samples were incubated in dibenzyl ether (DBE; without shaking) until cleared (overnight) and then stored in DBE at room temperature before imaging.

### LSFM and stereomicroscope imaging in mice

Cleared samples were imaged in transverse orientation with an LSFM (Ultramicroscope II, LaVision Biotec) equipped with a sCMOS camera (Andor Neo) and a 4×/0.3 objective lens (LaVision Biotec). Version 144 of Imspector Microscope controller software was used. The microscope chamber was filled with DBE. We used single-sided 3-sheet illumination configuration, with fixed x position (no dynamic focusing). The light sheet was generated by LED lasers (OBIS) tuned to 561 nm, 100 mW and 639 nm, 70 mW (LVBT Laser module 2nd generation). The light-sheet numerical aperture was set to 0.03. We used the following emission filters: 595/40 for Alexa Fluor 568 or 555, 680/30 for Alexa Fluor 647, and 830/780 for Alexa Fluor 790. Stacks were acquired using 4.5-μm z steps and a 30-ms exposure time per step, with an Andor CMOS sNEO camera. The 2× optical zoom was used for an effective magnification of 8×, 0.8 µm/pixel. Mosaic acquisitions were done with a 10% overlap on the full frame. Fluorescent stereo micrographs were obtained with AxioZoom.V16 fluorescence stereo zoom microscope (Carl Zeiss) equipped with an ORCA-Flash 4.0 digital sCMOS camera (Hamamatsu Photonics) or an OptiMOS sCMOS camera (QImaging).

### LSFM image processing and analysis

For display purposes, a γ correction of 1.47 was applied on the raw data obtained from the light-sheet fluorescent microscope. Images acquired with Imspector acquisition software in TIFF format were converted with Imaris File Converter to IMS files. Mosaics were reconstructed with Imaris stitcher, and then Imaris software (Bitplane, http://www.bitplane.com/imaris/imaris) was used to generate the orthogonal projections of data shown in all figures, perform area segmentation on a stack of image slices, and produce videos.

### Paraffin section immunolabeling and imaging

Vertebrae were dehydrated through ethanol, cleared in xylene, and embedded in paraffin. Serial cross sections (5 µm thick) were immunostained with rabbit anti-mouse LYVE1 (1:100) polyclonal antibody (11-034; AngioBio Co.). 3,3′-diaminobenzidine staining was performed using the biotin avidin complex kit (PK-6100; VectastainVector). Masson’s trichrome staining was carried out using the Masson Trichrome Kit (BioGnost; MST-100T; BioGnost). Hematoxylin (5 s) was used for counterstaining. HRP-labeled paraffin sections were analyzed with a Zeiss Axio Scope.A1.

### Flow cytometry analysis of LN immune cells

90 min after spinal intrathecal injection of OVA-A^488^, mice were anesthetized with ketamine/xylazine. LNs (mandibular, accessory mandibular, deep cervical, sciatic, and lumbar) were dissected and processed as previously described (Geraldo et al., 2021). LNs were digested with DMEM containing 2.5 mg/ml collagenase D and 5 U/ml DNase I for 20 min at 37°C. The digested tissue was passed through a 40-μm nylon cell strainer (Falcon), and red blood cells were lysed (Red Blood Cells Lysis buffer; Merck). After blocking with mouse FcR Blocking Reagent (MACS Miltenyi Biotec), single-cell suspensions were incubated with anti-CD45 BUV805 (clone 30-F11; BD), anti-CD11b BV421 (clone M1/70; BD), and anti-CD11c APC (clone N418; BD) antibodies. As a control, cells were stained with the appropriate isotype control. Data acquisition was performed on BD LSRFortessa X20, and analysis was performed with FlowJo_V10.

### MRI of dural and neck vasculature in humans

Imaging was performed with an MRI 3.0 T Magnetom Skyra (Siemens Healthineers). The following sequences were used before gadobutrol injection: Coronal Whole Brain 3D FLAIR (Coronal 3D acquisition; field of view, 256 mm^2^; 192 contiguous 1-mm slices; repetition and echo time [TR/TE], 5,000/375 ms; acquisition time, 6 min); Whole Brain T1 SPACE DANTE acquisition (Sagittal 3D acquisition, field of view 256 mm^2^; 224 contiguous 0.80-mm slices; TR/TE, 7,000/22 ms; acquisition time, 6 min). The following sequences were performed in all patients after gadobutrol (0.1 mmol/kg body weight, i.v., NDC 50419-325-12; Bayer Health Care) injection: Contrast-enhanced MR venography with elliptical-centric technique (3D sagittal acquisition; field of view 250 mm^2^; 208 contiguous 0.70-mm slices; TR/TE, 3.57/1.36 ms; acquisition time, 6 min); Coronal Whole Brain 3D FLAIR (Coronal 3D acquisition; field of view, 256 mm^2^; 192 contiguous 1-mm slices; TR/TE, 5,000/375 ms; acquisition time, 6 min); Whole Brain T1 SPACE DANTE acquisition (Sagittal 3D acquisition; field of view, 256 mm^2^; 224 contiguous 0.80-mm slices; TR/TE, 7,000/22 ms; acquisition time, 6 min). Eight patients were scanned with a 3D TOF sequence before gadobutrol injection.

### MRI postprocessing

#### 3D reconstructions of structures of interest

3D-Slicer platform (https://www.slicer.org) was used for semiautomated signal intensity–based thresholding and segmentation of the native sequences. Accordingly, dural lymphatics, perisinusal enhancement, and cLNs were extracted based on the T1-weighted post-gadobutrol SPACE DANTE sequence. The venous system was extracted based on the contrast-enhanced MR venography with elliptical-centric technique. The brain was extracted based on the FLAIR sequence, and the internal carotid arteries were extracted based on either the 3D TOF sequence before gadobutrol injection or the T1w-SPACE DANTE post-gadolinium sequence. Fusion of the 3D reconstructions of the dural venous sinuses and MLVs confirmed that lymphatic vessels were not misdiagnosed as small slow-flow veins and allowed detailed analysis of veno-lymphatic relationships.

#### Volumetric quantification

Volumes were measured in mm^3^ with the segment statistics module and Labelmap statistics by two blinded experienced neuroradiologists. All volumes including MLVs, dural venous sinuses, and brain volumes were normalized against the total intracranial volume (TIV). We used the module extension SwissSkullStripper (http://www.slicer.org/wiki/Documentation/Nightly/Modules/SwissSkullStripper) to automatically extract and measure the TIV by postprocessing the 3D T1 SPACE DANTE sequence. Normalized volumes were expressed as the ratio ([volume/TIV] × 10^−6^). Total MLV volume was defined as the volume of MLVs covering the superior sagittal sinus, the straight sinus, and both lateral sinuses including the confluence of sinuses. Lateral sinus–related MLVs were defined as the MLVs covering both lateral sinuses and the confluence of sinuses as well. Straight- and superior sagittal-related MLVs were defined as the MLVs covering the straight sinus and the superior sagittal sinus, respectively, without the confluence of sinuses. Finally, CAV-related MLVs were defined as the MLVs covering the CAV, without neighboring MLVs along the trigeminal nerves and the inferior petrosal sinuses. Because the dural venous sinuses have very different volumes, we normalized the MLV volume against their related venous sinus volume using the MLV/vein ratio to compare the lymphatic coverage between dural sinuses.

### Statistics

No statistical methods were used to predetermine sample size. Five to six mice were analyzed by experimental group (*n* = 5–6 mice/group). The investigators were blinded during experiments and outcome assessment.

Statistical data analysis was performed with Prism 6.0 software (GraphPad). For discrete variables (immune cell %), data are presented as mean SEM. A two-tailed, unpaired Mann–Whitney *U* test was done to determine statistical significance between two groups. For comparison between more than two groups, one-way ANOVA was performed, followed by Dunn’s multiple comparison test. Differences were considered statistically significant if the P value was <0.05 (*, P < 0.05; **, P < 0.01).

For quantitative MRI, ICC was calculated to assess observer agreement for volume measurements. ICC was classified as poor (0–0.20), fair (>0.20–0.40), good (>0.40–0.75), or excellent (>0.75). Correlations between quantitative values were assessed with nonparametric Spearman correlation tests.

### Graphic design

Illustration software was used to create Fig. 1 A and Fig. 6 A (BioRender.com). 3D-schemas of Figs. 1 E, 2 A, 3 A, 4 A, 4 B, 5 H, 5 N, 8 A, and S3 A as well as Videos 4 and 8 were generated using 3D-Blender, a free and open-source 3D computer graphics software toolset, from horizontal, sagittal, and coronal representations of the rat dural venous anatomy (Scremin, 2004) and from horizontal, sagittal, and coronal head sections labeled with vascular markers.

### Online supplemental material

Fig. S1 shows CSF tracer distribution after ICM and intraspinal injection. Fig. S2 shows LSFM imaging of OVA-A^555^ tracer lymphatic drainage and OVA-A^555^ accumulation around dural sinuses. Fig. S3 shows LSFM imaging of CSF drainage through the cribriform plate and inside the nasal cavity. Fig. S4 describes sacral spinal cord outflow. Fig. S5 shows meningeal and skull vascular MRI in humans. Videos 1, 3, 5, 6, 2, and 7 are coronal (1, 3, 5, 6) and sagittal (2, 7) LSFM videos of clarified half-heads from mice sacrificed 45 min after intrathecal injection of OVA-A^555^ into the caudal spine. Samples 4–6 were immunolabeled with anti-LYVE1 antibodies. Videos 4 and 8 are 3D schematics of the mouse meningeal blood vasculature and the cranial venolymphatic system, respectively.
